# Environment and diet shape the geography-specific *Drosophila melanogaster* microbiota composition

**DOI:** 10.1128/aem.00883-25

**Published:** 2025-09-25

**Authors:** Joseph T. Gale, Rebecca Kreutz, Sarah J. Gottfredson Morgan, Emma K. Davis, Connor Hough, Wendy A. Cisneros Cancino, Brittany Burnside, Ryan Barney, Reese Hunsaker, Ashton Tanner Hoyt, Aubrey Cluff, Maggie Nosker, Chandler Sefcik, Eliza Beales, Jack K. Beltz, Paul B. Frandsen, Paul Schmidt, John M. Chaston

**Affiliations:** 1 Department of Plant and Wildlife Sciences, Brigham Young University 6756, Provo, Utah, USA; 2 Department of Biology, University of Pennsylvania 6572, Philadelphia, Pennsylvania, USA; Norwegian University of Life Sciences, Ås, Norway

**Keywords:** latitude, temperature, photoperiod, neutral theory, lactic acid bacteria, local adaptation, life history, rapid adaptation

## Abstract

Geographic and environmental variation in the animal microbiota can be directly linked to the evolution and wild fitness of their hosts, but it can be difficult to explain the underlying patterns. Here, we sought to better understand wild variation in the microbiota composition of *Drosophila melanogaster*. First, environmental temperature predicted geographic variation in fly microbial communities more consistently than latitude did. The microbiota also differed between wild flies and their diets, supporting previous conclusions that the fly microbiota is not merely a reflection of diet. Flies feeding on different diets varied significantly in their microbiota composition, and flies sampled from individual apples were exceptionally depauperate for the lactic acid bacteria (LAB), a major bacterial group in wild and laboratory flies. However, flies bore significantly more LAB when sampled from other fruits or compost piles. Follow-up analyses revealed that LAB abundance in the flies uniquely responds to fruit decomposition, whereas other microbiota members better indicate temporal seasonal progression. Finally, we show that diet-dependent variation in the fly microbiota is associated with phenotypic differentiation of fly lines collected in a single orchard. These last findings link covariation between the flies’ dietary history, microbiota composition, and genetic variation across relatively small (single-orchard) landscapes, reinforcing the critical role that environment-dependent variation in microbiota composition can play in local adaptation and genomic differentiation of a model animal host.

**IMPORTANCE:**

The microbial communities of animals influence their hosts’ evolution and wild fitness, but it is hard to predict and explain how the microbiota varies in wild animals. Here, we describe that the microbiota composition of wild *Drosophila melanogaster* can be determined by temperature, humidity, geographic distance, diet decomposition, and diet type. We show how these determinants of microbiota variation can help explain lactic acid bacteria (LAB) abundance in the flies, including the rarity of LAB in some previous studies. Finally, we show that wild fly phenotypes segregate with the flies’ diet and microbiota composition, illuminating links between the microbiota and host evolution. Together, these findings help explain how variation in microbiota compositions can shape an animal’s life history.

## INTRODUCTION

Animal-associated microorganisms (“microbiota”) can profoundly impact the behavior, physiology, and evolution of their hosts. The types and abundances of microorganisms in wild animals, especially animals colonized by horizontally acquired poly-species communities, often vary dramatically in response to numerous factors, including diet, time, and space. Because the types and abundances of the microorganisms can determine specific host traits, these changes in microbiota composition can influence the adaptation of their hosts (1). Here, we sought to better understand the causes of variation in the microbiota composition of animals in the wild by studying the microbiota of the fruit fly *Drosophila melanogaster*, a host with a relatively low-diversity and low-abundance microbial community that is a model for understanding patterns of microbial community assembly (2), microbe-microbe interactions (3, 4), and host-microbe interactions, including in a wild setting (1, 5–7).

As in many other animals with a horizontally acquired gut microbiota, the *D. melanogaster* microbiota composition is driven by the diet, the host, and other members of the community. In the wild or the laboratory, a single fly typically bears several hundred thousand bacterial cells from fewer than 100 species, most of which are consolidated in fewer than 10 highly abundant species (8–17). Flies are typically dominated by acetic acid bacteria (AAB), lactic acid bacteria (LAB), or bacteria from the order Enterobacteriaceae (5, 6, 8–10, 12, 15, 18–20), and the types and abundances of these microorganisms are typically influenced by the same factors as influence the gut microbiota in mammals: diet (15, 16, 20), host genotype (21), and individuality, including vial effects for flies reared in the same containers (16, 22). In the laboratory, members of the *Acetobacter*, *Levilactobacillus*, and *Lactiplantibacillus* genera have been commonly used as representative isolates in the flies (23). Studies with bacteria from these and other genera have revealed that some, but not all, bacterial strains persistently colonize the flies for longer than the bulk passage of diet through the gut, and there are specific foregut niches for some of these persistently colonizing bacteria (22, 24–28). Also, the types and abundances of the microbes in the flies are distinct from those in the diet (16), dramatically influenced by host genetic selection and microbe-microbe interactions (3, 21, 23, 29, 30), and typically best defined by neutral community assembly rules, suggesting strong roles for ecological drift and passive dispersal (6, 12). Finally, LAB can have singularly critical roles on their hosts, including by provisioning branched chain amino acids and other molecules to combat undernutrition (8, 31), modulating the immune protection through signaling and gut acidification (3, 32), establishing a colonization-compatible environment for other members of the microbiome (27), and sending olfactory-guided signals to their hosts that determine foraging decisions (33). Thus, a substantial body of work has established key drivers of microbial community structure and how certain members can shape host traits and life history.

The fruit fly *D. melanogaster* is an established model for studying geographic variation and the microbiota. The geographic life history of *D. melanogaster* may be the most extensively studied of any animal on the planet, with hundreds of studies documenting latitudinal clines in allele frequencies at candidate genes (34–38) and fitness-associated traits (39–43), and in patterns of genomic differentiation (44–46). Populations of *D. melanogaster* in the eastern United States show clear genomic and phenotypic differentiation between different latitudes and seasons. The microbiota composition of *D. melanogaster* also varies across these geographic and seasonal clines and can profoundly influence the life history traits and evolution of its host (1, 5, 6). Two recent studies of the microbiota of wild flies from the eastern USA have each reported substantial geographic variation in microbiota composition, with some conflicting findings between the studies. In one of these studies, we reported substantial numbers of LAB in many of the sampled flies and a latitudinal gradient in the AAB:LAB ratio, and suggested that this pattern revealed congruence between microbial abundance, the influence of those microorganisms on host traits, and the host traits naturally adopted in the sampled locations (5). Another study sampled deeply at more locations and found essentially no LAB in any of the samples and little evidence for a latitudinal cline within or between host variation in microbiota composition (6). The authors concluded that variation in microbiota composition was determined primarily by neutral processes and strict host filtering. Also, following evidence that LAB are rare in some (6, 15) but not other (5, 12, 18–20) samplings of wild flies, the authors suggested that LAB may be more likely to colonize laboratory than wild flies. These disparate findings show gaps in our understanding of what determines the wild fly microbiota composition.

To better understand the relationship between sampling location and *D. melanogaster* microbiota composition, we asked four major questions: (i) Can previously observed latitudinal patterns in microbiota composition be observed in fresh samplings of wild flies? (ii) What is the relationship between the microbiota of wild flies and their wild diet? (iii) Why are LAB readily recovered in some, but not all, wild *D. melanogaster* samplings? (iv) How is variation in the microbiota composition of wild flies related to their life history? We addressed these questions by comparing the sequencing results of previous and new collections of wild *D. melanogaster*, measuring the microbiota composition of wild and laboratory fly populations reared on distinct environmental conditions or on different diets, and experimentally dissecting the contributions of time and diet decomposition to microbiota composition. We also measured a key life history trait in wild-caught, laboratory-reared fly populations. Together, these results provide evidence that specific environmental conditions predict patterns in microbiota composition better than latitude, that diet influences but does not necessarily seed the wild fly microbiota, and that fly phenotypes can segregate with the diet of flies in the wild.

## RESULTS

### A geography-specific *D. melanogaster* microbiota composition is associated with environmental temperature

**Fig 1:**
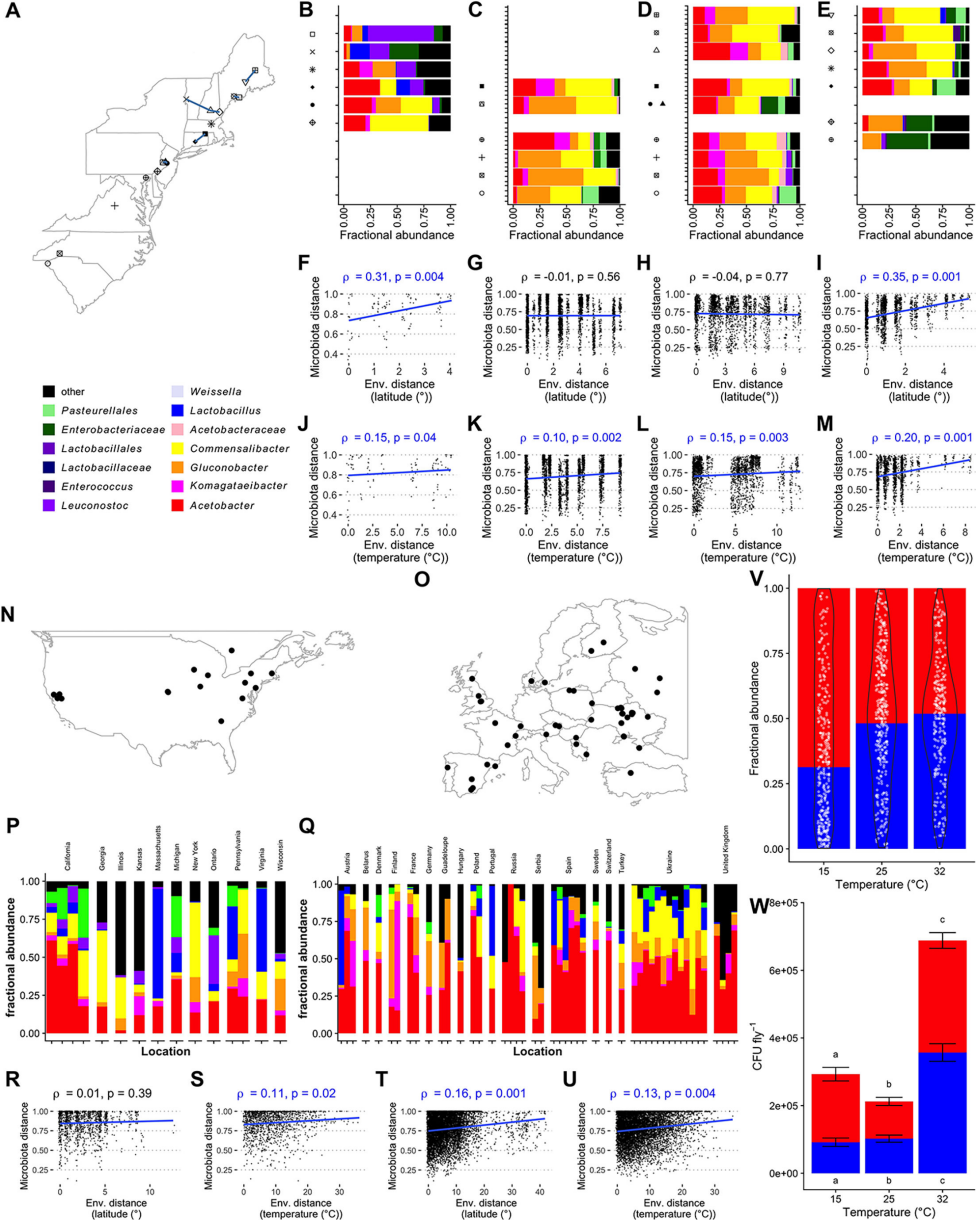
Latitude- and temperature-dependent variation in microbiota composition of *D. melanogaster* from the eastern USA in multiple years and samplings. *D. melanogaster* from (**A**) the same general area in the eastern USA was sampled and sequenced across portions of the 16S rRNA V4 (**A and E**) or V1–V2 (**C and D**) regions by two laboratories in (B) 2009 (apples and peaches, *N* = 15), (**C**) 2018 (grapes, *N* = 71), (**D**) 2018 (apples, *N* = 79), and (**E**) 2021 (apples, *N* = 60). Taxon plots are an average of samples, which are pools of flies (B, rarefaction threshold [rt] = 475) or individual flies (**C–E**, rt = 500). Mantel tests reporting the relationship of the Bray-Curtis and environmental distances between each sample were also performed, with environmental distance calculated from distance matrices based on (**F–I**) latitude or (**J–M**) latitude plus maximum temperature and minimum relative humidity (RH) on the day of sampling. Letters over F–M report the Mantel test correlation coefficient ρ and the *P*-value results when the results are (blue) or are not (black) significant. (**N–U**) A similar analysis as for eastern USA flies was conducted using sequences extracted from whole-genome sequencing of flies collected with the DEST data set. (**N and O**) Maps, (**P and Q**) taxon plots, and (R–U) Mantel test results for DEST flies from (**N, P, R, and S**) North America and (**O, Q, T, and U**) Europe are reported. Samplings from Guadeloupe, a department of France in the Caribbean, are not shown on the map. (**V and W**) Common garden populations of individual isofemale lines from each location in Fig. S6A were reared under six-species gnotobiotic conditions in the laboratory until adult flies were 3 days old, transferred to test conditions for 3 days, then the microbiota composition of pools of two surface-sterilized adults was analyzed by homogenization and dilution plating (two pools of two females and two males per vial, three vials per common garden population in each experiment, and three separate experiments in time). The relative (V) and absolute (W) abundances of AAB (red) and LAB (blue) CFUs in flies reared at varying temperatures. Relative abundances are shown as the mean of AAB counts divided by the mean of LAB counts, with the fraction of LAB shown as a white point and the overlayed violin plot showing the distribution of fractional LAB abundance. Significant differences in relative abundances of LAB were determined by PERMANOVA of CFU counts rarefied to 10,000 CFUs per sample (Table S2). Absolute CFU abundances are shown as the mean and SEM of all replicates. Significant between-group differences in absolute AAB and LAB abundances are shown as determined by a Kruskal-Wallis test with a post-hoc Dunn test, and different letters over (AAB) or under (LAB) the bars report significant differences in their abundance.

We reanalyzed two previously published samplings of flies in the eastern USA and compared these to the results of freshly collected samples to better understand the reasons for their different outcomes. The previous studies included samples collected in 2009 (Fig. 1B, from apples and peaches) and 2018 (Fig. 1C [from grapes] and Fig. 1D [from apples]). We added two additional samplings, one in the eastern USA (Fig. 1E, 2021, from apples) and one in Utah, USA (Fig. S1, 2020, from peaches). Each eastern USA collection included some sites shared with the other studies (Fig. 1A; Fig. S2). The most abundant genera in the new samplings mirrored the previous studies, including that the flies were dominated by AAB, with substantial Enterobacteria or LAB in some samples. Mantel tests of the relationship between microbiota composition and latitude recapitulated previous conclusions by showing that 2009, but not 2018, microbiota composition significantly covaried with latitudinal distance (Fig. 1F through H; Fig. S3) (5, 6). As in 2009, microbiota composition of the new samplings covaried with latitude (Fig. 1I; Fig. S1C). We sought to reconcile the different studies’ outcomes by testing whether environmental conditions could help explain the variation in microbiota composition from different locations. Of 41 variables we tested, the microbiota in all five locations significantly covaried with just one factor—the daily maximum temperature (Table S1)—suggesting temperature could be a major determinant of wild fly microbiota composition (Fig. 1J through M; Fig. S1D). This conclusion mirrors a previous study that examined samplings of *Drosophila* across Europe, though it compared a smaller number of environmental variables (47). Daily maximum temperature and latitude were not significantly correlated in most of these samplings, suggesting that temperature was not just a surrogate measure of latitude (Fig. S4A through E).

We then tested if these findings applied to flies sampled in other areas and at various times by comparing the same environmental and geographic metrics with the microbiota composition of flies collected as part of the Drosophila Evolution over Space and Time (DEST) data set, which were collected across North America and western Europe mostly between 2014 and 2016 (Fig. 1N through Q; [48]). As before, the geographic and microbiota distances covaried significantly in one, but not both, sampling area (Fig. 1R and T). However, the difference in maximum temperature on the day of sampling was significantly correlated with the distance in microbiota composition in samples from both North America and Europe (Fig. 1S and U; Table S1). We also confirmed experimentally that variation in temperature significantly determines gnotobiotic microbiota composition (Fig. 1V and W; Table S2; Fig. S5). Beyond temperature, UV irradiance and humidity also covaried with microbiota composition in many samplings (Table S1), although they were usually not significantly correlated with each other (Fig. S4F through J). We investigated microbiota effects of humidity previously (49) and report in the present work that photoperiod is also associated with significant changes in microbiota composition, though the changes are less marked than those caused by temperature (Fig. S6; Table S2). From these findings, temperature emerges as the most consistent—though not the only—driver of variation in wild *D. melanogaster* microbiota composition.

### The *D. melanogaster* microbiota is not a copy of its dietary microbiota

**Fig 2:**
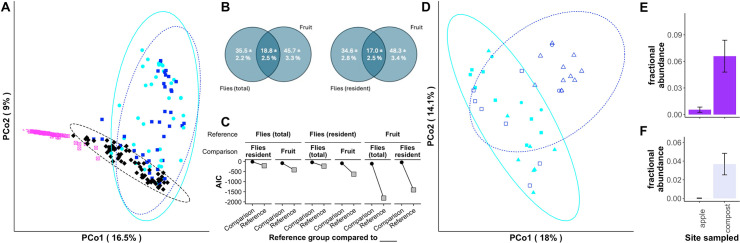
The total and resident *D. melanogaster* microbiota composition is distinct from the diets of wild flies. (**A**) Principal coordinates ordination of Bray-Curtis dissimilarity distances for the microbiota of individual wild *D. melanogaster*, their diets, and nearby soil locations from multiple locations in the eastern USA. Flies were immediately frozen after collection (“flies [total]”) or starved in empty vials for >2 h (“flies [resident]”) after transient microorganisms had passed through the fly gut with the bulk flow of diet. The OTU table was rarefied to 500 reads per sample. (**B**) Venn diagrams showing the average fractional abundance of ASVs ± s.e.m. that were unique to or shared between sampled fruit and the total or resident fly microbiota. (**C**) AIC values for neutral models calculated with the group indicated in the “reference” row were compared to itself (“Reference,” ■) or the group in the “comparison” (●) row. (**D**) Principal coordinates ordination of Bray-Curtis dissimilarities for flies collected from individual apples or compost piles at multiple locations in the eastern USA, rarefied to 845 reads per sample. Reads assigned to the genera (**E**) *Leuconostoc* and (**F**) *Weissella* differed significantly in abundance between locations as determined by ANCOM.

To further show how the environment can determine the fly microbiota, we compared the microbiota between flies with a total (resident + transient) or resident-only microbiota, neighboring fruits and soils. We defined the resident microbiota as the microorganisms present in flies after >2 h of starvation in empty fly vials, allowing sufficient time for the bulk flow of diet to pass through the flies (50). Microbiota composition varied with both the sample type (fly [total or resident], fruit, or soil, *F*_3,218_ = 20.08, *R*^2^ = 0.20, *P* < 10^−4^; Table S3) and the orchard they were sampled from (*F*_7,218_ = 2.28, *R*^2^ = 0.05, *P* < 10^−4^; Table S3). Community dissimilarity was comparable between fruits and flies with a total or resident-only microbiota, and all were distinct from soils (Fig. 2A; Fig. S7A through C). More than 85% of all sequencing reads were assigned to the <20% of amplicon sequence variants (ASVs) shared between fruit and flies. Among these, *Commensalibacter*, *Lactobacillus*, *Enterococcus*, and reads that could not be assigned below the *Pasteurellales* level were all significantly more abundant in flies than in diets (Fig. 2B; Table 1). Other bacterial strains were more abundant in the diet than the flies (Table 1), together identifying differences between the microbiota of flies and their diets and classifying bacteria from the different genera as fly- or diet-preferred. No bacterial genera were differentially abundant between total microbiota- or resident-only flies. When we compared the microbiota of flies and their diets using a Sloan neutral model, we found better evidence to support that the fly microbiota is neutrally assembled from other flies than from the diet because neutral model Akaike information criterion (AIC) scores were lowest when the sampling source was other flies, not diets (Fig. 2C). This outcome suggests that the flies’ microbiota is not merely a reflection of the diet, but other deterministic factors, possibly including temperature and humidity as identified above, contribute to variation in microbiota composition. Together, these findings show that the microbiota of wild flies and their environment, including diet, is distinct. They also help refute the notion that the fly microbiota is just a sampling of the diet, which refutation has been well-supported previously (22, 24, 25).

**TABLE 1:** Read counts of bacterial genera in flies relative to the fruits on which they were feeding^*a*^

Genus	Flies (total microbiota)				Flies (resident microbiota)				
	Flies	Fruit	% in fly	*P*-value	Flies		Fruit	% in fly	chisq_p
*Commensalibacter*	6,113	0	100	10^−99^	6,675		23	100	10^−99^
*Lactobacillus*	n.t.				255		2	99	10^−54^
*Enterococcus*	n.t.				416		18	96	10^−79^
Unassigned *Enterobacteriaceae*	2,009	1,187	63	10^−43^	1,474		1,683	47	0.0004
Unassigned *Pasteurellales*	641	393	62	10^−13^	550		290	66	10^−17^
*Pseudomonas*	92	100	48	0.62	61		189	24	10^−14^
*Gluconobacter*	3,484	3,878	47	0.00004	3,839		3,680	51	0.10
*Acetobacter*	2,418	4,565	35	10^−121^	2,342		3,923	37	10^−74^
*Gluconacetobacter*	1,120	2,574	30	10^−113^	489		2,932	14	10^−99^
Unassigned *Xanthomonadaceae*	62	193	24	10^−15^	91		213	30	10^−11^
Unassigned *Bacteria*	88	1,420	6	10^−246^	59		1,991	3	10^−99^
*Lactococcus*	5	274	2	10^−56^	9		356	2	10^−71^
Unassigned *Proteobacteria*	1	393	0	10^−84^	n.t.				

^*a*^ n.t. = not tested, <1% of total reads collected.

Even though the fly microbiota is not a copy of its diet, the diet can still influence the flies’ microbiota composition. Indeed, the resident microbiota of flies collected from compost at the three sites where we found such piles differed from the resident microbiota composition in flies collected from fallen apples (*F*_1, 31_ = 2.11, *R*^2^ = 0.05, *P* = 0.01; Table S4; Fig. 2D; Fig. S7D). The two genera that differed in abundance in flies from these sites were both members of the LAB: *Leuconostoc* and *Weissella* (Fig. 2E and F). These findings show diet-dependent variation in microbiota composition of flies from the same orchard and suggest that flies sampled from apples are less likely to bear LAB abundantly, with a shift from 1.4% (Fig. 1D) or 4.8% (Fig. 1E) of reads assigned to Lactobacillales in flies sampled exclusively from apples, relative to 8.0%–8.1% when flies were sampled from peaches or compost (Fig. S6D; Table S5). This latter finding may help explain why recent fly collections—both from apples—detected few LAB (Fig. 1D and E). However, because the compost piles had many different types of decomposing material and were not just apples, it is not clear if the differences in fly microbiota composition are from the substrate or its decomposition state.

### Wild diets determine differences in the *D. melanogaster* microbiota composition

**Fig 3:**
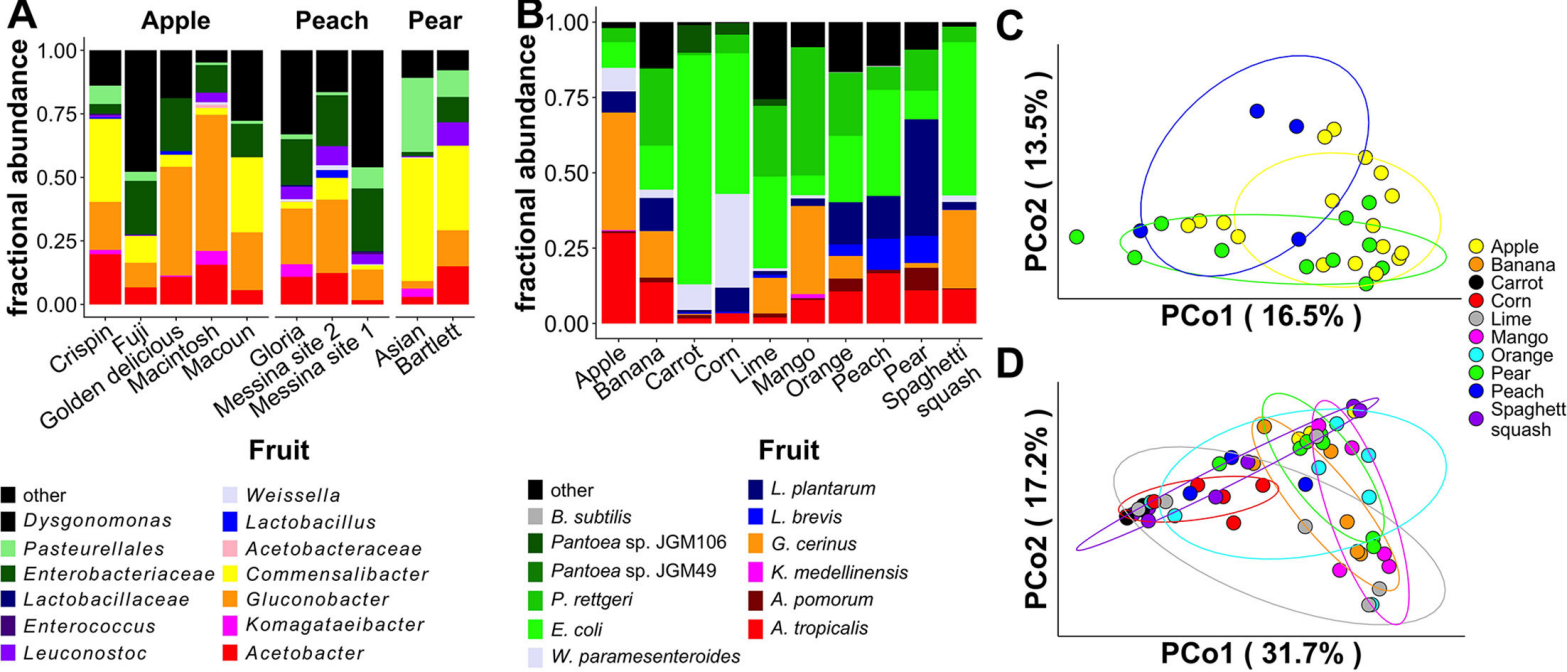
The microbiota composition of wild and laboratory *D. melanogaster* varies with diet type. We sequenced the 16S rRNA V4 region of the *D. melanogaster* microbiota collected from different fruits in the wild and the laboratory. (**A and C**) Composition of the resident microbiota in wild male *D. melanogaster* collected from fallen fruit at Lyman Orchards in Middlefield, CT. (**A**) Bars are the averages of multiple samples, each rarefied to 120 reads (*N* = mean 3.2 ± sem 0.25, min = 2, and max = 4 samples per bar). (**C**) Principal coordinate ordination of Bray-Curtis dissimilarity distances. (**B and D**) Composition of the total microbiota in gnotobiotic 12-sp *D. melanogaster* CantonS male flies. (**B**) Bars are the averages of multiple samples, each rarefied to 200 reads (*N* = mean 5.3 ± sem 0.54, min = 3, and max = 7 samples per bar). (**D**) Principal coordinate ordination of Bray-Curtis dissimilarity distances. Clustering confidence was set to 0.7 to draw ellipses.

To follow up on the role of diet in shaping the fly microbiota composition, especially the abundance of LAB, we compared the microbiota composition of flies sampled from different wild diets in a single orchard. The flies’ microbiota significantly varied when sampled individually from varieties of apple, peach, and pear in a single orchard (*F*_2_, _31_ = 2.04, *R*^2^ = 0.10, *P* < 10^−4^; Fig. 3A and C; Table S6). However, no bacterial groups at any taxonomic level passed the analysis of the composition of microbiomes (ANCOM) significance threshold, preventing us from attributing the community-level variation to specific taxa. We adopted a more controlled approach by showing that the microbiota composition of laboratory flies reared under gnotobiotic conditions with 12 different bacterial species varied significantly with the type of fruit on which they were reared (*F*_9_, _52_ = 2.42, *R*^2^ = 0.34, *P* < 10^−4^; Fig. 3B and D; Table S7). Four of the species varied significantly in abundance in flies reared on the different fruits: *Levilactobacillus brevis*, *Weissella paramesenteroides*, *Gluconobacter cerinus*, and *Pantoea* sp. JGM106 (ANCOM *W* score = 11 for each species). *L. brevis* was particularly prevalent in peaches, pears, and oranges, and LAB also tended to have higher average abundance in the non-apple samples from our wild samplings (Fig. 3A), exemplified by LAB representing 20.1% of the total sampled community (Table S5). Additionally, apples had a greater average AAB abundance than any other samples (Fig. 3B), consistent with observations that flies sampled from apples in the USA east coast had a lower relative abundance of LAB than our samplings from peaches in Utah, USA (Fig. 1D and E; Fig. S1; Table S5). These findings reveal that the *D. melanogaster* microbiota varies with diet in both the wild and laboratory, including in flies reared with the same starting set of microorganisms.

### Population dynamics of the wild *Drosophila* microbiota are distinct with time and decomposition of wild fly diets

**Fig 4:**
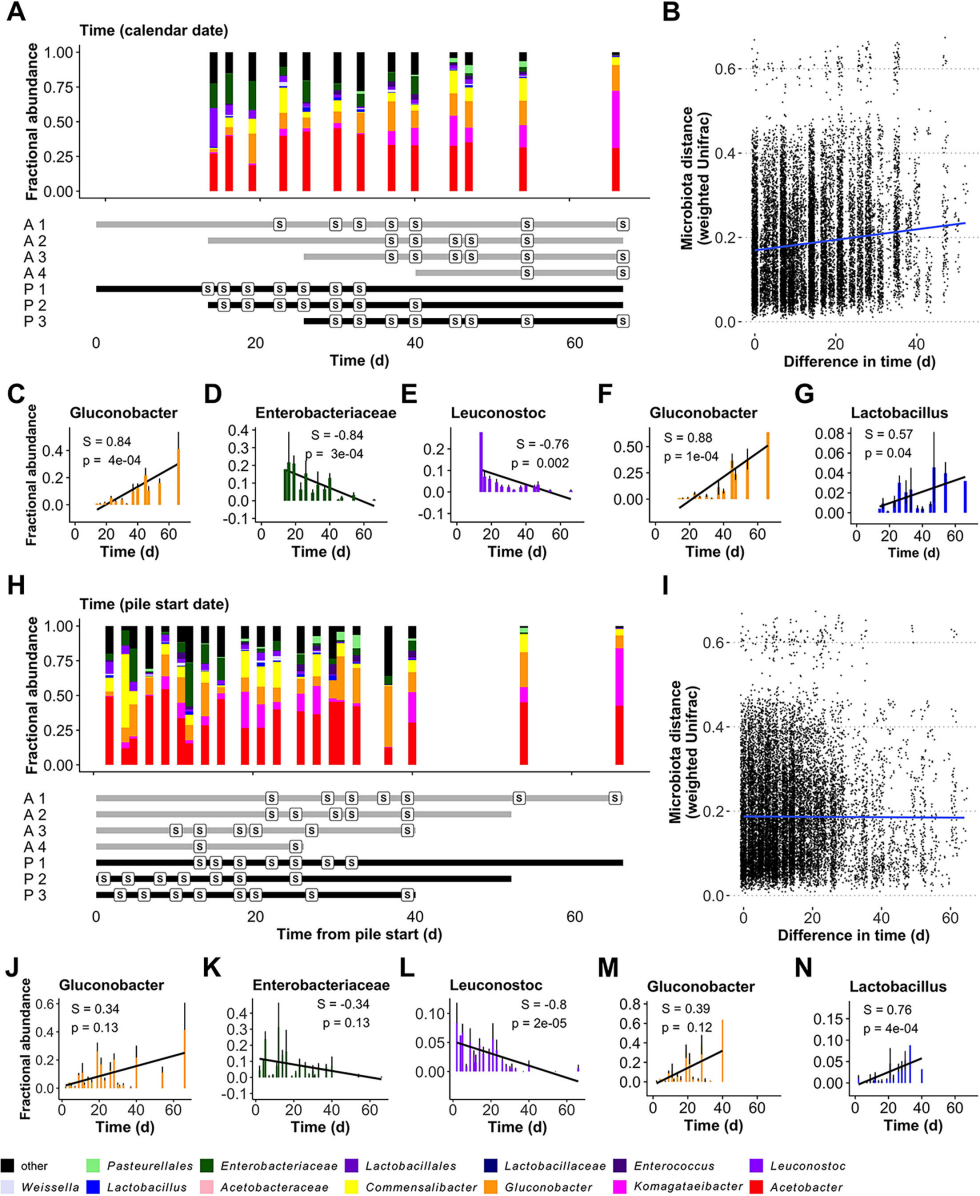
The microbiota composition of wild *D. melanogaster* varies with time and diet decomposition. We sequenced the 16S rRNA V4 region of the resident microbiota in individual wild *Drosophila* collected from 1-bushel piles of fruit established at different times in an experimental orchard in Provo, UT. The same data are shown on two timescales, either relative to (**A–G**) calendar date (time = 0 when the first piles were established) or (H–N) pile establishment time (time = 0 for each pile when it was established). (**A and H**) Taxon plot, plus a timeline showing the establishment and sampling times (S) from each of four apple (A) and three peach (P) piles. (**B and I**) Mantel test showing the relationship of the weighted Unifrac and calendar date (**B**) or pile establishment time (**I**) distances between each sample, including the slope of the trendline (blue). Plots showing the abundances of specific members of the microbiota that varied in (**C–E and J–L**) apples and peaches, or only in peaches (**F, G**, **M, and N**) in one or both timescales. The Spearman’s rank correlation coefficient (S) and *P*-value (p), plus a trendline for change in abundance over time (black line), are shown for each. Samples were rarefied to 500 reads before processing.

We next investigated how time and diet decomposition shape the microbiota composition of wild flies, especially their LAB abundance, by capturing flies from fruit piles in different decomposition states. We established new piles of peaches and apples every 2 weeks and sequenced the resident microbiota of flies sampled twice weekly from the various piles. The microbiota of the flies varied with the type of fruit they were sampled from (*F*_1, 183_ = 11.79, *R*^2^ = 0.05, *P* < 10^−4^; Table S8) and with time (*F*_1, 183_ = 10.5, *R*^2^ = 0.05, *P* < 10^−4^; Table S8; Fig. 4A). Also, the microbial communities grew more different from each other when sampled at distant times (Fig. 4B; Fig. S8, Mantel test statistic *s* = 0.12, *P* < 10^−4^). Turning to individual taxa, *Gluconacetobacter*, *Leuconostoc*, and reads that could not be assigned below the family Enterobacteriaceae significantly varied over time, and each was significantly correlated with time (Fig. 4D and E). No genera varied significantly in flies sampled only from apples, but *Gluconacetobacter* and *Lactobacillus* reads varied significantly with time in flies sampled from peaches (Fig. 4F and G).

Then, we tested if diet decomposition shaped microbiota composition when time was defined relative to the “establishment time” of the pile instead of calendar date. There was a significant effect of establishment time (Fig. 4H, *F*_1, 183_ = 4.06, *R*^2^ = 0.02, *P* = 0.01) on microbiota composition but no correlation with establishment time (Fig. 4I; Fig. S8, *s* = −0.005; *P* = 0.50). Furthermore, *Gluconacetobacter* (peaches and apples, Fig. 4J; peaches only, Fig. 4M) and *Enterobacteriaceae* (Fig. 4K) reads that covaried with calendar date did not covary with the establishment time of the piles. Conversely, *Leuconostoc* abundance in peaches and apples (Fig. 4L) and *Lactobacillus* abundance in peaches only (Fig. 4N) varied more significantly with establishment time than calendar date. Together, these findings suggest that the relative abundance of most of the genera that vary in abundance in the flies covaries with time, but the populations of the two LAB that varied across the different samplings responded more to the establishment time of the piles, or their decomposition, than calendar date. The absence of these patterns when apples were considered alone is consistent with our previous observations that LAB are depauperate in flies feeding on apples and shows that some diets are incompatible with the seasonal patterns we document here. Together, these experiments reveal that variation in the abundance of LAB in the flies is complex and determined at least in part by interactions between time, diet decomposition, and microbial compatibility with the fly diet.

### Diet determines microbiota-dependent genetic stratification of *D. melanogaster* in a single orchard

**Fig 5:**
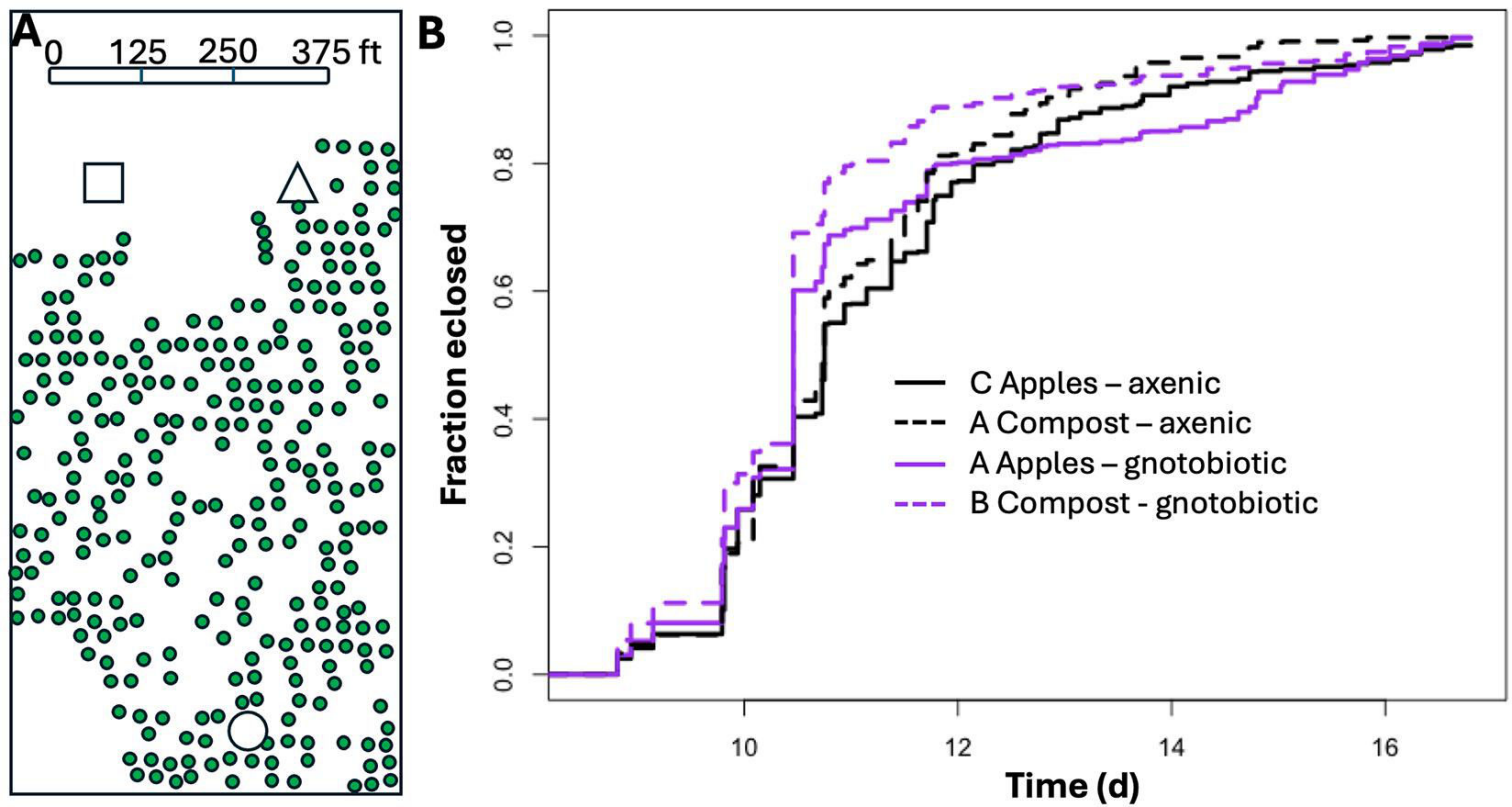
*D. melanogaster* phenotypes segregate with the diet from which they are collected. (**A**) Wild *D. melanogaster* was sampled from a compost pile (□) or individual fallen apples directly under an apple tree (○, ∆) throughout a single orchard, shown in a digital recreation of an Apple maps screenshot taken in Summer 2024. Green circles represent apple tree positions in the sampled orchard. (**B**) Isofemale lines derived from the wild collections were reared in the laboratory under gnotobiotic or bacteria-free conditions, and their development time to adulthood (eclosion) was measured. Significant differences between groups were determined by a Cox mixed-effects model and are shown as compact letter displays in the legend.

Finally, we tested if diet-dependent variation in microbiota composition was associated with variation in the life history of flies in a wild setting. We reasoned that since the microbiota is an agent of selection that can drive adaptation of its host (1), flies from diets with distinct microbiota composition might segregate phenotypically. When we measured the development time to adulthood for wild-caught isofemale lines reared in the laboratory under gnotobiotic and bacteria-free conditions, there were significant, non-interactive effects of the dietary source (*Z*_1, 4173_ = 8.04, *P* < 10^−15^) and rearing condition (*Z*_1, 4173_ = 7.81, *P* < 10^−14^) of the flies (Fig. 5A and B, non-significant interaction was *Z*_3_, _4173_ = −1.54, *P* = 0.12). The phenotypic pattern was that the flies in the more LAB-rich conditions (the compost piles) developed to reproductive maturity more quickly than flies sampled from LAB-depauperate, apple, conditions in multiple locations throughout the orchard (Fig. 2E and F). Together, these findings support the conclusion that diet-dependent microbiota composition is associated with the genotypic differentiation in two distinct fly populations sampled from the wild.

## DISCUSSION

We sought here to define patterns in the microbiota composition of wild flies and reconcile differences between conflicting previous analyses. We found that latitudinal differentiation in microbiota composition was more consistently explained by considering temperature than latitude. Follow-up experiments in the laboratory confirmed that temperature and, to a lesser extent, photoperiod influence microbiota composition (a role for humidity had been previously demonstrated [49]). Additionally, we provide evidence that even though the flies’ microbiota is not a direct reflection of their diet, the type and decomposition of the fly diet influence distinct members of the fly microbiota. Whereas the AAB was prevalent and abundant in a variety of diets, LAB was depauperate in flies sampled from apples and grapes (0.5%–4.8% relative abundance) but had higher relative abundances in our analyses that included non-apple substrates as diet (5.6%–31.4%; Table S5). Together, these factors can help explain the varied differences in the microbiota composition of wild-caught flies and show that numerous factors, most of which vary asynchronously with time and space, help determine variation in microbiota composition.

Diet explains some of the patterns in microbiota abundance observed across four different fly samplings (Fig. 1; Fig. S1). LAB was nearly absent in flies sampled from individual apples (Fig. 1D and E) and grapes (Fig. 1C) but was abundant when sampled from rotten peaches (Fig. S1) or not explicitly from individual fruits (Fig. 1B). We did not analyze the microbiota of gnotobiotic flies reared on grapes, but gnotobiotic flies reared on apples bore the highest fraction of AAB of any fruit we sampled, whereas those reared on peaches bore relatively high LAB loads. The wild samplings suggest that grapes, like apples, support relatively high AAB loads, and the absence of LAB from the more recent east coast fly samples may have been driven by sampling from fruits that naturally support high levels of AAB (Fig. 1C through E). If samplings from individual apples or grapes, qualitatively assessed to be rotten by the authors (6), are less decomposed than a compost pile, then decomposition state may also contribute to these differences. However, this is not possible to ascertain post-hoc. Still, diet type alone cannot explain differences in microbiota composition between experiments because Fig. 1B flies were sampled from peaches and apples, and the peach-sampled flies had intermediate LAB abundance relative to the apple samples. If the higher latitude samplings were from more decomposed fruits (this was not recorded at sampling), it could help explain why there were more LAB in flies collected from apples at high latitudes than in peaches at lower latitudes. Aside from studies that included laboratory inoculations or sampled flies from unrecorded diets, our data overall suggest that a range of 5%–10% is a good estimate for the relative abundances of LAB in wild flies. This number may underestimate overall LAB abundances; however, as most of the samples reported here used a DNA extraction protocol that lacks a lysozyme digestion, we have observed lower than expected counts of gram-positive strains when sequencing DNA extracted from whole-cell positive controls for microbiome analysis (unpublished data). As the effects should be consistent across samplings, this should not alter the overall conclusions reported above, but may suggest the 5%–10% as a lower, rather than an upper, limit, for LAB abundance. Regardless, these findings show that controlling for diet decomposition state and diet type in fruit flies provides context for variation in microbiota composition, and our approaches highlight one way that key drivers of microbiota variation can be identified in wild sampled animals.

Even though diet and its decomposition state led the wild fly microbiota composition to vary, the microbiota composition of wild flies and their proximal diets at the time of collection were generally incongruent. The most abundant taxa in flies were usually detected in the diet, and vice versa, but multiple approaches—comparison of ASV identities, modeling if fruit was the source of the fly microbiota, and directly comparing composition between sample types—all failed to link the abundance of those ASVs between the two sample types. The conspicuous difference between the microbiota of flies and neighboring fruits was that *Commensalibacter* was the most abundant ASV in flies and essentially absent from the diet. *Lactobacillus* and *Enterococcus* were also abundant in flies but not their diets. These findings are consistent with the substantial body of work showing that laboratory and wild flies can be colonized persistently by specific sets of microorganisms (22, 24, 27, 30, 51). It remains unclear what the reservoir of the fly- or diet-specific microorganisms is and how each is transferred between flies or to fresh diets, but it is evident that abundant fly microbiota ASVs are not necessarily sampled commonly from their diets. The low abundance and prevalence of the fly-preferred microbes in the flies’ diets also suggest transmission fidelity between flies. Alternatively, flies may have obtained their microorganisms from diets distinct from those that they were feeding on at the time of capture; however, the finding that the microbiota differed between flies feeding on different diets in the same orchard is inconsistent with this expectation. Because the fly microbiota is assembled neutrally (6, 12, 52) and has priority effects (27), flies are likely either colonized early with microorganisms that are not abundant in their diet, or certain microorganisms are adept at invading established communities. Together, these findings are consistent with previous demonstrations that the host and community interactions drive the microbiota composition in the gut and the external fly environment (16, 30, 49).

Along with host genotype and diet, the abiotic environment also shapes *Drosophila* microbiota composition. Whereas latitude inconsistently predicted microbiota composition, the microbiota of flies sampled from different diets or years was individually correlated with environmental factors that influence microbiota composition. Temperature was the most consistent covarying factor. Elevated temperatures increased the absolute and relative abundance of LAB in flies, consistent with their higher maximum growth temperatures than AAB. The tolerance of LAB to heat may also explain their elevated abundance in compost piles, which can reach internal temperatures above 60°C, relative to individual fallen fruits. High frequencies of LAB at warmer temperatures are inconsistent with our previously published expectation to detect LAB more abundantly in flies at high latitudes (5) but may possibly be explained by differences in the decomposition state of the diet. Finally, the relatively weak correlation coefficients for temperature show that a substantial portion of microbiota variation remains explained, likely at least in part from other environmental factors we describe here. Some of these may be nested with latitude, especially since latitude and temperature were not correlated with each other in the different samplings (Fig. S4).

Temperature, photoperiod, humidity, diet type, and diet decomposition state vary asynchronously with season in different geographic areas, raising questions about the role of seasonal variation in microbiota composition. A recent analysis showed that the microbiota of flies in outdoor mesocosms varies with seasonal progression, including a summer-to-fall shift from *Acetobacter* to *Commensalibacter* dominance, and a mid-summer peak in the abundance of *Wautersiella*, a genus that was not abundant in our analyses (7). These seasonal patterns were independent of diet because the flies were fed the same laboratory diet throughout the experiment. We have not performed experiments to determine if the strong covariation of microbiota composition with temperature reflects or causes the seasonal change in the microbiota. We note that established patterns in the seasonal evolution of *D. melanogaster*, along with the ability to track how variation in microbiota composition influences fly adaptation, suggest an ideal framework for future investigation of the relationships between seasonal variation, local adaptation of a host, and variation in microbiota composition.

The data presented here suggest that fly evolutionary history segregates with diet and diet-dependent microbiota composition. We interpret that diet and microbiota composition contribute phenotypic structure to wild populations even in the presence of expected gene flow. The wider differentiation in developmental periods of gnotobiotic than axenic flies is consistent with previous demonstrations that the microbiota can enhance phenotypic variance in wild fly populations (5), though our laboratory experiments likely underrepresent the potential variance as they were conducted with standardized diets and microbiota. The causes of diet-dependent segregation of host phenotypes are unknown, but they do not result from genetic selection on the microbiota (Fig. S9), which can occur in some populations as described in reference 21. Instead, phenotypic differences between wild flies may be driven by diet, sampling-specific, or mediated directly by host genotype based on genetic determinants of, e.g., dispersal, dietary, or feeding preference traits (33, 53–58). We observed that flies feeding on compost in the wild, which promotes LAB abundance (Fig. 2E and F), have a shorter developmental period than flies collected from apples (Fig. 5B), a diet that restricts LAB prevalence and abundance (Fig. 2E and F). The negative relationship between LAB abundance and development traits is surprising because LAB is usually associated with longer, not shorter, development times of their *Drosophila* hosts in laboratory experiments (5, 59). There are several possible explanations for finding the shorter development times of flies exposed to more LAB. LAB, especially *Lactiplantibacillus plantarum*, can accelerate the developmental period of larvae reared in undernutritional conditions, relative to axenic flies (8, 31, 32, 60), and the dietary condition of the compost relative to nutritionally rich or limited laboratory diets is not known. Also, we cannot rule out species-specific effects since we did not characterize the influences of the bacterial strains from the flies we collected. A third possibility is that flies exposed to more LAB are under compensatory evolutionary selection and evolve faster host-programed development times to maintain a target fitness phenotype in the wild. This latter idea would suggest relatively long-term partitioning of the hosts, diets, and microbes, for which we have no evidence at this time. Seasonal selection experiments in outdoor mesocosms, an approach adopted by us and by others in the past (1, 7), would provide one avenue to investigate this latter possibility. Regardless, we have shown here that conditions with different fly microbiomes are associated with changes in a model host’s evolved traits.

Our analysis does not focus on the prevalent intracellular bacterium *Wolbachia*, which manipulates the reproduction, physiology, and life history of many insects, including *Drosophila* (61–69). Most of our data sets were poorly balanced for the presence and absence of *Wolbachia*, so formal comparisons between *Wolbachia-*colonized and *Wolbachia*-free samples would be confounded by small sample sizes. However, we consistently checked for *Wolbachia* colonization status (±) in sequencing data, reported it as a covariate when significant, and included comparisons of the *Wolbachia-*colonized and *Wolbachia*-free flies where appropriate in supporting information. As an exception, the fly samples from Fig. 3A were well balanced for *Wolbachia* status. Among these, *Wolbachia*-positive flies bore more family-level *Acetobacteraceae* reads and, at the ASV level, more of a *Commensalibacter* ASV than *Wolbachia*-free flies (Fig. S10). The positive relationship between *Commensalibacter* and *Wolbachia* is somewhat surprising because a sister genus, *Acetobacter*, has been reported by us and others to be negatively associated with *Wolbachia* abundance (1, 65, 70). However, it is not unprecedented, given a previously demonstrated interaction between *Wolbachia* and *Commensalibacter* that influences the flies’ life history (7). These findings highlight the importance *Wolbachia* plays as a part of the *Drosophila* microbiota, and the recent use of outdoor enclosures to study *Wolbachia-Drosophila*-microbiota interactions highlights an elegant way to do so in a native setting while also maintaining a balanced statistical design.

In summary, we reveal order in the *D. melanogaster* microbiota composition by considering multiple underlying factors. Variation in diet type and decomposition can help explain why LAB, commonly dominant in laboratory flies, is nearly absent in some recent fly microbiota surveys. Changes in LAB abundance also have possible implications for diversity in life history patterns of flies at a single location. These efforts model the possibility to explain and make sense of geographic variation in microbiota composition, with its links to animal life history.

## MATERIALS AND METHODS

### General rearing and culture conditions

We reared flies at 25°C on a 12 h light:dark cycle. Flies from wild collections were reared on a molasses diet (5). All other experiments used a *Wolbachia-*free CantonS fly line from Mariana Wolfner, reared on a yeast-glucose diet (23).

### Wild fly collections

We collected samples from eight orchards in the eastern USA in Fall 2021 and five orchards in Utah, USA, in Fall 2020 (Table S10). At each location, we sampled 10–20 individual fallen fruit sites to collect: the fruit, neighboring soil, a male *D. melanogaster* fly bearing a total microbiota, and a male *D. melanogaster* fly bearing resident microbiota. At each site, 10–20 flies were captured from an aerial insect net and divided equally into two empty, sterile vials: one immediately frozen on dry ice; the other left for >2 h for defecation of non-resident microorganisms, then frozen on dry ice. Fruit and soil were sampled to 1/2-inch depth using a 1/4-inch (fruit) or 1/2-inch (soil) diameter fruit corer that was pre-sterilized in 10% bleach for 30 s then rinsed in sterile H_2_O. Each was immediately stored on dry ice. We also performed aerial collections of flies at compost piles in three locations (Table S10) and single-fruit collections for the resident fly microbiota from many different apple, peach, and pear varieties in the orchard at Middlefield, CT. All samples were stored on dry ice until they could be permanently stored in a −80°C freezer. Live fly populations (Tables S10 and S11) were derived as isofemale lines from individual female flies, identified as *D. melanogaster* by morphological and molecular means (49), then propagated individually in the laboratory. Flies were not surface sterilized at any time during this process.

Locale-specific fly populations were established from individual isofemale lines by 2-3 generations of common-garden mixing as described previously (49). Eggs laid by F2 or F3 flies were reared as gnotobiotic flies in association with the six bacterial species *Acetobacter tropicalis* DmCS_006, *Acetobacter sp*. DsW_54, *Acetobacter sp*. DmW_125, *Lactiplantibacillus plantarum* DmCS_001, *W. paramesenteroides* DmW_115, and *Leuconostoc suionicum* DmW_098. Two days after adult flies eclosed, vials were transferred to new conditions to measure the influence of a 3-day perturbation in photoperiod (left at a 12 h light:dark cycle or incubated at 1 h light:23 h dark or 23 h light:1 h dark) or temperature (left at 25°C or moved to 15°C or 32°C) on the adult fly microbiota. Three days later, we measured the adult fly microbiota. Flies caught from compost and apples in the same orchard were reared under axenic and gnotobiotic conditions as individual isofemale lines, not in a common garden.

### Axenic and gnobiotic fly rearing and enumeration of bacterial colonization

Embryos laid by *D. melanogaster* were collected, dechorionated in 10% bleach for two 150 s washes, and rinsed three times with sterile water, and 30–60 embryos were transferred to 7.5 mL sterile molasses diet in a 50 mL microcentrifuge tube. Separately, bacterial strains were cultured overnight in de Man-Rogosa-Sharpe (MRS) medium (Criterion C5932) for 1–2 days at 30°, normalized to OD_600_ = 0.1, and mixed in equal ratios. Then, we inoculated 50 µL of the bacterial mixture into the sterile eggs and allowed the eggs to develop to adulthood to derive gnotobiotic embryos; or no bacteria were inoculated to eggs that were left to develop as axenic, or bacteria-free, flies. When the adult flies were 4–7 days old, the adult fly microbiota was measured by surface-sterilizing flies in 70% ethanol while under light CO_2_ anesthesia, performing whole-body homogenization, and dilution plating the homogenate for CFU counting as described previously (71). From each fly vial, we collected four pools of two male flies and four pools of two female flies and surface-sterilized them by brushing with 70% ethanol, then allowing residual ethanol to air dry. Fly homogenates were serially diluted onto MRS (to culture AAB and LAB) and MRS plus 10 µg/mL chloramphenicol and 10 µg/mL erythromycin (to culture only AAB, all gnotobiotic AAB strains are resistant, and all gnotobiotic LAB strains are susceptible). All dilutions and plating were performed using an EpMotion 96. Plates were incubated at 30°C for 2–3 days, and the antibiotic-free plates were cultured in sealed CO_2_-flooded containers. Colonies of LAB and AAB were manually or automatically counted (71) to compare the abundances of each bacterial order in the fly microbiota. Each experiment was repeated three times with triplicate vials for each population and condition. CFU count data were analyzed using permutational analysis of variance (PERMANOVA) as described previously (49). We used PERMANOVA instead of some other test (e.g., assuming normality when the data were not normal, transforming the data, or performing a nonparametric test) because our CFU count data were not normally distributed and because the data had many underlying fixed or random effects (source location of the host flies, location, treatment condition [such as temperature], vial, and experimental-replicate-in-time). It also allowed us to examine both absolute (using raw counts) and relative (using rarefied counts) differences using the same underlying test. Briefly, significant influences of treatments on microbiota composition were determined by PERMANOVA of a Bray-Curtis beta-diversity distance matrix calculated from an operational taxonomic unit (OTU) table we manually constructed from the LAB and AAB CFU count data. We used custom scripts, QIIME2, and the R package Vegan (72) in these steps, and PERMANOVA was always performed with 1,000 permutations. For absolute abundance data, the raw counts in the OTU table were used. When a significant difference in community composition was detected, we interpreted this to indicate a difference between LAB and AAB abundances with the corresponding effect, as these were the only two members of the community, and we performed a Kruskal-Wallis test with a post-hoc Dunn test and the R packages dunn.test (73), rcompanion (74), and multcomp (75) on each individual member (AAB or LAB) to determine how they varied with treatment. For relative abundance data, the Bray-Curtis distance matrix was calculated from an OTU table rarefied to 10,000 (temperature) or 4,000 (photoperiod) counts.

### Sample preparation and 16S rRNA gene sequencing

In the laboratory, DNA was extracted from each sample using the Zymo Quick-DNA Fecal/Soil Microbe 96 Kit (D6011) in 50 µL elution buffer: individual flies, first examined under a microscope for morphological confirmation as male *D. melanogaster* by absence of a distinct genital arch; 0.02 g of soil; and 0.05 g of fruit. Each 96-well plate included four controls: a positive extraction control (Zymo D6300), a negative reagent-only control, and two empty wells for PCR amplification controls.

We performed 16S rRNA V4 marker gene sequencing using a previously described dual-barcoding approach (76), including a negative control with no template and a positive control (Zymo D6331) for each 96-well plate. Negative controls did not yield visible bands by gel electrophoresis. We normalized the reactions using the Just-a-Plate normalization kit (Charm Biotech, JN-120-10) and combined equivalent volumes of 96 samples into single pools. The pools were concentrated using a Zymo gDNA Clean and Concentrator 11-302C kit, fragment size distributions were evaluated on an Agilent FemtoPulse (Agilent Technologies, Santa Clara, CA, USA), and fragments in the 250–450 bp range were selected on a Sage Science Blue Pippin (Sage Science, Beverly, MA, USA). The final molarity of the pool was estimated via quantitative PCR at the BYU DNA sequencing center. Then, we combined the pools and sequenced them on an Illumina MiSeq using 500 cycle v2 chemistry as described previously (76).

### Sequence analysis

Demultiplexed sequence reads were analyzed using QIIME2 (77) and R 4.1.2 (78). Previously published data are from PRJNA589709 and PRJNA873107. Using DADA2 (79), reads were denoised and dereplicated, and ASVs were called using trimming lengths that maximize quality scores of the reads. Taxonomic assignments to ASV were made using the GreenGenes classifier 13_8_99 (80), and samples were assigned as *Wolbachia* positive if 20% or more of the total reads were assigned to *Wolbachia*. In the figure legends, taxonomic assignments to “Gluconacetobacter” were manually renamed as “Komagataeibacter.” Then, reads assigned to *Archaea*, *Chloroplast*, *Mitochondria*, or *Wolbachia* were discarded. OTU tables were filtered to various thresholds per sample, reported in each corresponding figure legend. Significant differences between groups were determined by PERMANOVA (81) of beta-diversity distance metrics (82, 83). Phylogenetic trees, supporting the use of the Unifrac distance metrics, were built with fasttree2 (84) based on mafft alignment (85). ANCOM was used to define significant differences in the abundances of individual microbes between samples (86). Beta-diversity distance analysis, calculation of Venn diagrams (87), Spearman’s rank correlation tests, and PERMANOVA (81) were all performed in R. Neutral models were applied to sequencing data in R as described previously (88, 89), by providing taxon plot matrices to the “fit_sncm()” function of the tyRa package (90). Each matrix was constructed from all samples corresponding to the same substrate (starved flies, unstarved flies, fruits, or soils) across the analysis, and the “pool” argument was used to specify the source from which each test matrix was derived. This function provided us with AIC values for each test. Sloan neutral models compare abundances of microbial taxa between a source and a target community to identify microorganisms whose abundance in a source and prevalence in a target do or do not correspond. To perform Mantel tests in R, environmental metadata for each location were downloaded from the USA and Americas (1998–2022) database at https://nsrdb.nrel.gov/data-viewer in 30- or 60-minute intervals or from the Meteostate Prime Meridian: Africa and Europe database in 60-minute intervals. Maximum, average, or minimum values of each character were calculated on the day of sampling. A distance matrix was then constructed based on individual or multiple values and compared to a beta-diversity distance matrix produced as described above. Mantel tests used Spearman’s rank correlations. Sequences from the DEST data set were downloaded from the SRA using fasterq-dump v.2.10.8 from the SRA toolkit (https://github.com/ncbi/sra-tools), and microbial profiling was performed using MetaPhlan3 and the ChocoPhlan v30 database (91). The OTU table obtained from all outputs was then analyzed using Bray-Curtis distance metrics as described above.

### Gnotobiotic fly rearing on fruit diets

To measure the impact of distinct diets on the microbiota composition of *D. melanogaster*, we reared gnotobiotic *D. melanogaster* CantonS on 10 types of fruit obtained from grocery stores in Provo and Orem, UT. We diced, froze, and transferred 5–10 g of each fruit type to a 50 mL centrifuge tube, then autoclaved the tubes. Then, we transferred 30–60 bleach-sterilized, dechorionated CantonS fly embryos to the diets as described above, and inoculated each vial with a mixture of separately cultured and OD_600_ = 0.1-normalized bacterial community composed of *Acetobacter pomorum* DmCS_004, *Acetobacter tropicalis* DmCS_006, *Bacillus subtilis* 168, *Escherichia coli* K-12 MG1655, *G. cerinus* Dm-58, *Komagataeibacter medellinensis* NBRC 3288, *Lactiplantibacillus plantarum* DmCS_001, *L. brevis*, *Pantoea* sp. JGM49, *Pantoea dispersa* JGM106, DmCS_003, *Providencia rettgeri* JGM232, and *W. paramesenteroides* DmW_115. When the flies were 5- to 7-day-old adults, one pool of five male flies was collected from each vial, frozen at −80°C, and the V4 region of the 16S rRNA gene was sequenced and analyzed as described above. We performed four separate experiments, each with triplicate vials of flies, to target the collection of three pools of flies in each of three experiments. However, recovery of adult flies was often challenging because some diets poorly supported fly growth or were wet and adult flies drowned. We collected and sequenced as many samples as we could recover from the four experiments.

### Wild fly rearing on decomposing diets

To dissect the separate influences of seasonal progression and diet decomposition on microbiota composition, we established fresh fruit piles in a field site in Provo, UT, in August 2022. Each pile was established by dropping one bushel of fruit (Allred’s Orchards, Payson, UT) from a height of 4 feet onto a grass lawn. We established piles from apples and peaches every 2 weeks for 6 weeks and a fourth apple pile 2 weeks later (fresh peaches were no longer available). Twice a week, up to 30 fruit flies were sampled from each pile with a hand insect vacuum into empty vials, starved for 2 h, and then frozen in a −80°C freezer. Sampling continued until hard freeze onset, November 2022, and the 16S rRNA V4 region on individual flies was sequenced as described above. Only 7 of 146 male flies were assigned as *D. simulans* by morphological examination, so we analyzed male *D. melanogaster* and female *Drosophila* together in this experiment.

### Measuring fly development time to adulthood

Fly development time to reproductive maturity was measured at 1, 6, and 11 h into the daily light cycle each day as the time to eclosion for each individual pupa on the side of a fly vial until all flies in a vial had eclosed or there were two consecutive time points where no flies eclosed, whichever came first. Significant differences in fly development time were determined using a Cox mixed-effects survival model with the source diet (apples or compost) and rearing treatment (axenic or gnotobiotic) as interactive terms and experimental replicate (four fly vials per treatment were reared in each of three distinct experiments in time) as a random effect. The analyses were performed using survival-model-specific R packages (92, 93).

### Data analysis

Some R packages we used are not cited elsewhere (94–107). Maps were made in R (108). A screenshot from Apple Maps taken in summer 2024 was used as a template in Fig. 5A. Raw data and scripts for these analyses are available at https://github.com/johnchaston/Gale2024, most prominently the script file Gale_script.Rmd and the knitted HTML file from that script, Gale_script.html.zip. OTU tables and metadata corresponding to each microbiome data set are included in the Supporting Data Appendix.

## Data Availability

Sequences from this study are available in the NCBI Sequence Read Archive under BioProject accession PRJNA1306182 and BioSample accessions SAMN51281715:SAMN51281944, SAMN50641377:SAMN50641402, SAMN50629839:SAMN50629891, and SAMN50678895:SAMN50679078.

## SUPPLEMENTAL MATERIAL

Data S1 to S24 aem.00883-25-s0001.xlsx OTU tables and metadata for sequencing datasets.

Supplemental material aem.00883-25-s0002.pdf Tables S1 to S11; Fig. S1 to S10.

ASM does not own the copyrights to Supplemental Material that may be linked to, or accessed through, an article. The authors have granted ASM a non-exclusive, world-wide license to publish the Supplemental Material files. Please contact the corresponding author directly for reuse.
