# Supplementary material for: Environment and diet shape the geography-specific Drosophila melanogaster microbiota composition
Source: Appl Environ Microbiol. 2025 Sep 25;91(10):e00883-25. doi: 10.1128/aem.00883-25 (PMC12542676; doi:10.1128/aem.00883-25)

Table S1. Significant covariance by a Mantel test between a single value and Bray-Curtis distance in sequencing datasets. Cells contain Mantel test p-value. Bold represents $p < 0.05$.

Environmental parameter	*Sampling experiment* 2009	2018 Vineyard	2018 Orchard	2021	2020 Utah	DEST N. A.	DEST Europe
Daily maximum temperature	**0.003**	**0.002**	**0.041**	**0.001**	**0.007**	**0.019**	**0.004**
Daily minimum relative humidity	**0.003**	**0.001**	**0.021**	**0.001**	0.065	0.29	**0.012**
Minimum precipitable water	**0.001**	**0.002**	**0.022**	**0.002**	0.166	0.14	0.35
Daily maximum Clearsky direct normal irradiance	**0.003**	**0.001**	**0.024**	**0.033**	0.125	0.07	0.12
Daily average wind direction	**0.045**	**0.002**	**0.033**	**0.001**	0.089	0.60	0.24
Daily average relative humidity	**0.001**	**0.001**	0.053	**0.001**	0.223	0.20	**0.013**
Daily maximum Clearsky global horizontal irradiance	**0.029**	**0.041**	0.05	**0.011**	0.312	**0.016**	**0.002**
Daily minimum temperature	**0.001**	**0.015**	0.095	**0.001**	0.376	**0.038**	0.055
Daily maximum dew point	**0.001**	**0.003**	0.155	**0.045**	0.39	0.26	0.15
Daily average dew point	**0.015**	**0.001**	0.168	**0.002**	0.11	0.24	0.26
Daily average diffuse horizontal irradiance	**0.001**	**0.017**	0.182	**0.001**	0.471	0.94	0.21
Daily maximum diffuse horizontal irradiance	**0.011**	**0.001**	0.221	**0.006**	0.312	0.97	0.35
Daily maximum wind speed	**0.001**	**0.001**	0.225	**0.008**	**0.012**	0.061	0.11
Daily average windspeed	**0.002**	**0.001**	0.248	**0.045**	**0.029**	0.087	0.23
Daily minimum wind speed	**0.001**	**0.001**	0.354	0.054	0.19	0.18	0.39
Daily average temperature	**0.001**	0.059	0.071	**0.001**	0.591	**0.03**	**0.003**
Daily minimum wind direction	**0.001**	0.087	0.257	**0.014**	**0.029**	0.78	0.57
Daily minimum dew point	**0.001**	0.137	0.086	**0.001**	0.242	0.24	0.36
Daily average precipitable water	**0.001**	0.202	**0.013**	**0.005**	0.277	0.18	0.097
Daily average Clearsky direct normal irradiance	**0.015**	0.276	0.261	**0.016**	0.316	0.16	**0.005**
Daily maximum precipitable water	**0.035**	0.324	**0.03**	**0.001**	0.261	0.15	**0.037**
Daily maximum relative humidity	**0.021**	NA	0.064	**0.001**	0.513	0.27	0.74
Daily maximum Clearsky diffuse horizontal irradiance	0.058	0.07	0.206	**0.001**	0.069	0.69	0.62
Daily average Clearsky global horizontal irradiance	0.06	**0.028**	0.219	**0.005**	0.357	**0.046**	**0.001**
Daily average Clearsky diffuse horizontal irradiance	0.083	0.178	0.191	**0.001**	0.085	0.77	0.19
Daily average surface albedo	0.15	0.987	**0.043**	**0.002**	**0.013**	0.37	0.76
Daily maximum surface albedo	0.15	0.987	**0.043**	**0.002**	**0.013**	0.45	0.75
Daily minimum surface albedo	0.15	0.987	**0.043**	**0.002**	**0.013**	0.48	0.75
Daily average pressure	0.18	0.957	**0.045**	0.126	**0.008**	**0.026**	0.094
Daily maximum fill flag	0.247	**0.002**	0.279	**0.001**	0.143	0.53	0.79
Daily maximum pressure	0.248	0.879	0.133	0.114	**0.008**	**0.031**	0.08
Daily minimum pressue	0.303	0.94	**0.044**	0.131	**0.016**	**0.022**	0.09
Dailiy average solar zenith angle	0.491	**0.027**	0.359	**0.001**	0.437	**0.009**	**0.001**
Daily maximum solar zenith angle	0.577	0.531	0.168	**0.001**	0.329	**0.009**	**0.019**
Daily average fill flag	0.615	0.95	0.285	**0.002**	0.088	0.072	0.11
Daily minimum solar zenith angle	0.71	**0.003**	0.378	**0.001**	0.226	**0.019**	**0.001**
Daily maximum wind direction	0.988	0.566	**0.017**	**0.001**	**0.011**	0.16	0.20

Table S2 PERMANOVA results for CFU counts (AAB and LAB) of the fly microbiota (see Fig 1V, 1W)

	Absolute						***Relative***					
	**Df**[a]	**SS**[b]	**R^2**	**F**	**p**[c]		**Df**[a]	**SS**[b]	**R^2**	**F**	**p**[c]	
Geography	7	20.94	0.10	19.01	0.001	***	7	8.50	0.14	29.75	0.001	***
Temperature	2	15.45	0.07	49.10	0.001	***	2	4.81	0.08	58.90	0.001	***
Sex	1	0.46	0.00	2.93	0.03	*	1	0.12	0.00	2.99	0.07	.
Exp [d]	2	9.93	0.05	31.54	0.001	***	2	1.57	0.03	19.21	0.001	***
*G * T* [e]	13	6.36	0.03	3.11	0.001	***	13	1.56	0.03	2.93	0.001	***
*G * S*	7	1.00	0.00	0.91	0.60		7	0.39	0.01	1.36	0.20	
*T * S*	2	1.39	0.01	4.42	0.002	**	2	0.19	0.00	2.28	0.10	
E / Plate [f]	3	2.84	0.01	6.01	0.001	***	3	0.29	0.00	2.35	0.07	.
*G * T * S*	13	2.05	0.01	1.00	0.48		13	0.85	0.01	1.60	0.07	.
E / P / Vial [g]	81	35.37	0.16	2.78	0.001	***	81	10.98	0.19	3.32	0.001	***
Residual	762	119.92	0.56				738	30.10	0.51			
Total	893	215.71	1.00				869	59.33	1.00			
Geography	7	13.11	0.05	14.45	0.001	***	7	1.76	0.03	7.42	0.001	***
Photoperiod	2	1.50	0.01	5.77	0.001	***	2	0.68	0.01	9.99	0.001	***
Sex	1	0.20	0.00	1.53	0.181		1	0.04	0.00	1.28	0.253	
Exp [d]	2	17.35	0.07	66.90	0.001	***	2	10.69	0.16	157.70	0.001	***
*G * P* [e]	14	6.16	0.02	3.39	0.001	***	14	1.50	0.02	3.15	0.001	***
*G * S*	7	1.15	0.00	1.26	0.187		7	0.60	0.01	2.54	0.013	*
*P * S*	2	0.57	0.00	2.21	0.033	*	2	0.03	0.00	0.45	0.639	
E / Plate [f]	3	5.07	0.02	13.03	0.001	***	3	2.58	0.04	25.36	0.001	***
*G * P * S*	14	2.29	0.01	1.26	0.119		14	0.74	0.01	1.55	0.086	.
E / Plate / Vial [g]	141	59.31	0.23	3.24	0.001	***	141	10.31	0.15	2.16	0.001	***
Residual	1207	156.50	0.59				1144	38.78	0.57			
Total	1400	263.20	1.00				1337	67.71	1.00			

[a] degrees of freedom

[b] sum of squares

[c] p-value

[d] One of three separate experiments in time

[e] “*” = the interaction term

[f] “/” = the nesting term; Plate = the 96-well plate on which samples were homogenized from which they were dilution plated

[g] vial = the source vial the flies were grown in (30-50 mixed sex flies per vial)

Table S3 PERMANOVA results for 16S sequencing data corresponding to Figure 2A

	Unweighted Unifrac						Weighted Unifrac					Bray Curtis				
	Df[a]	SS[b]	R^2	F	p[c]		SS	R^2	F	p		SS	R^2	F	p	
Sample type[d]	3	19.01	0.37	45.97	0	***	8.65	0.36	44.33	0	***	17.9	0.2	20.08	0	***
Orchard	7	2.08	0.04	2.16	0	***	0.87	0.04	1.9	0.02	*	4.74	0.05	2.28	0	***
Individual fruit	66	10.03	0.19	1.1	0.15		4.48	0.19	1.04	0.35		21.45	0.24	1.09	0.04	*
*S * O[e]*	18	3.71	0.07	1.5	0	***	1.73	0.07	1.48	0.04	*	9.91	0.11	1.85	0	***
Residual	124	17.09	0.33	NA	NA		8.06	0.34	NA	NA		36.84	0.41	NA	NA	
Total	218	51.92	1	NA	NA		23.79	1	NA	NA		90.85	1	NA	NA	

[a] degrees of freedom

[b] sum of squares

[c] p-value

[d] Flies (total), flies (resident), fruit, or soil

[e] "*" = the interaction term

Table S4 PERMANOVA results for 16S sequencing data corresponding to Figure 2D

	Unweighted Unifrac						*Weighted Unifrac*					*Bray Curtis*				
	**Df**[a]	**SS**[b]	**R^2**	**F**	**p**[c]		**SS**	**R^2**	**F**	**p**		**SS**	**R^2**	**F**	**p**	
Fresh or compost	1	0.56	0.1	4.49	$<10^{-4}$	***	0.11	0.08	3.53	0.02	*	0.87	0.07	3.25	$<10^{-4}$	***
Orchard	2	0.44	0.08	1.78	0.03	*	0.12	0.08	1.89	0.1		1.71	0.14	3.2	$<10^{-4}$	***
*F * O* [d]	2	0.28	0.05	1.13	0.29		0.17	0.12	2.77	0.03	*	1.26	0.1	2.35	$<10^{-4}$	***
Residual	33	4.1	0.76	NA	NA		1.02	0.72	NA	NA		8.82	0.7	NA	NA	
Total	38	5.38	1	NA	NA		1.41	1	NA	NA		12.65	1	NA	NA	

[a] degrees of freedom

[b] sum of squares

[c] p-value

[d] "*" = the interaction term

Table S5 Percentage of reads assigned to Lactobacillales in different samplings

Analysis	*% of reads assigned to Lactobacillales*	*Characteristic*
Figure 1B	31.4%	Wild flies sampled from unrecorded diets
Figure 1C	0.5%	Wild flies sampled from grapes
Figure 1D	1.4%	Wild flies sampled from apples
Figure 1E	4.8%	Wild flies sampled from apples
Figure 1P	26.9%	Wild flies sampled from unrecorded diets
Figure 1Q	9.4%	Wild flies sampled from unrecorded diets
Figure 2A / Figure S7B	1.8%	Wild flies samples from apples, apples, soil
Figure 2D / Figure S7D	8.0%	Wild flies sampled from apples and compost
Figure 3A	5.6%	Wild flies sampled from apples, peaches, and pears
Figure 3B	20.1%	Gnotobiotic flies sampled in the laboratory from different autoclaved diets
Figure 4	7.8%	Wild flies sampled from a time course of rotting apple and peach piles
Figure S1B	8.1%	Wild flies sampled from peaches

Table S6 PERMANOVA results for 16S sequencing data corresponding to Figure 3A,C

	Unweighted Unifrac						*Weighted Unifrac*					*Bray Curtis*				
	**Df**[a]	**SS**[b]	**R^2**	**F**	**p**[c]		**SS**	**R^2**	**F**	**p**		**SS**	**R^2**	**F**	**p**	
Fruit type[d]	2	0.43	0.08	1.36	0.13		0.12	0.10	2.41	0.04	*	1.08	0.10	2.04	$< 10^{-4}$	***
Wolbachia	1	0.32	0.06	2.00	0.03	*	0.14	0.11	5.32	$< 10^{-4}$	***	0.56	0.05	2.11	0.01	*
F / Variety[e]	7	1.34	0.25	1.20	0.15		0.37	0.29	2.07	0.02	*	2.69	0.26	1.45	0.01	*
*F * W*[f]	2	0.21	0.04	0.68	0.9		0.08	0.06	1.59	0.16		0.86	0.08	1.62	0.01	*
*(F / V) * W*	5	0.75	0.14	0.94	0.58		0.21	0.17	1.67	0.09		1.55	0.15	1.17	0.19	
Residual	14	2.23	0.42	NA	NA		0.36	0.28	NA	NA		3.71	0.36	NA	NA	
Total	31	5.28	1.00	NA	NA		1.29	1.00	NA	NA		10.46	1.00	NA	NA	

[a] degrees of freedom
[b] sum of squares
[c] p-value
[d] apples, peaches, or pears
[e] "/" = the nesting term
[f] "*" = the interaction term

Table S7 PERMANOVA results for 16S sequencing data corresponding to Figure 3B,D

	Unweighted Unifrac					*Weighted Unifrac*					*Bray Curtis*				
	**Df**[a]	**SS**[b]	**R^2**	**F**	**p**[c]	**SS**	**R^2**	**F**	**p**		**SS**	**R^2**	**F**	**p**	
Fruit	9	0.6	0.21	1.24	0.08	0.53	0.34	2.42	$<10^{-4}$	***	5.16	0.36	2.67	$<10^{-4}$	***
Residual	43	2.3	0.79	NA	NA	1.05	0.66	NA	NA		9.24	0.64	NA	NA	
Total	52	2.89	1	NA	NA	1.58	1	NA	NA		14.4	1	NA	NA	

[a] degrees of freedom

[b] sum of squares

[c] p-value

Table S8 PERMANOVA results for 16S sequencing data corresponding to Figure 4A, Figure 4H

	Unweighted Unifrac						Weighted Unifrac					Bray Curtis				
	Df[a]	SS[b]	R^2	F	p[c]		SS	R^2	F	p		SS	R^2	F	p	
Fruit type	1	0.96	0.03	6.64	$<10^{-4}$	***	0.18	0.04	8.72	$<10^{-4}$	***	3.21	0.05	11.79	$<10^{-4}$	***
Calendar date	1	0.78	0.03	5.41	$<10^{-4}$	***	0.22	0.05	10.5	$<10^{-4}$	***	3.03	0.05	11.15	$<10^{-4}$	***
Fly sex	1	0.43	0.01	3	$<10^{-4}$	***	0.04	0.01	1.68	0.12		0.69	0.01	2.53	$<10^{-4}$	***
Wolbachia	1	0.26	0.01	1.8	0.06		0.05	0.01	2.36	0.05		0.49	0.01	1.81	0.03	
F / pile [d]	5	1.49	0.05	2.07	$<10^{-4}$	***	0.14	0.03	1.36	0.12		5.4	0.09	3.97	$<10^{-4}$	***
*F * C* [e]	1	0.5	0.02	3.45	$<10^{-4}$	***	0.02	0	0.74	0.56		0.97	0.02	3.57	$<10^{-4}$	***
*(F / P) * C*	5	1.08	0.04	1.5	0.02	*	0.2	0.05	1.94	0.04	*	3.2	0.05	2.36	$<10^{-4}$	***
Residuals	168	24.17	0.81	NA	NA		3.53	0.81	NA	NA		45.7	0.73	NA	NA	
Total	183	29.67	1	NA	NA		4.38	1	NA	NA		62.7	1	NA	NA	
Fruit type	1	0.96	0.03	6.64	$<10^{-4}$	***	0.18	0.04	8.72	$<10^{-4}$	***	3.21	0.05	11.79	$<10^{-4}$	***
Establishment time	1	0.75	0.03	5.21	$<10^{-4}$	***	0.09	0.02	4.06	0.01	*	2.04	0.03	7.48	$<10^{-4}$	***
Sex	1	0.42	0.01	2.92	$<10^{-4}$	***	0.03	0.01	1.63	0.14	*	0.7	0.01	2.59	$<10^{-4}$	***
Wolbachia	1	0.22	0.01	1.54	0.11		0.04	0.01	2.06	0.07		0.42	0.01	1.54	0.08	
F / pile	5	1.57	0.05	2.18	$<10^{-4}$	***	0.29	0.07	2.72	$<10^{-4}$	***	6.46	0.1	4.75	$<10^{-4}$	***
*F * E*	1	0.5	0.02	3.45	$<10^{-4}$	***	0.02	0	0.74	0.56		0.97	0.02	3.57	$<10^{-4}$	***
*(F / P) * E*	5	1.08	0.04	1.5	0.02	*	0.2	0.05	1.94	0.04	*	3.2	0.05	2.36	$<10^{-4}$	***
Residuals	168	24.17	0.81	NA	NA		3.53	0.81	NA	NA		45.7	0.73	NA	NA	
Total	183	29.67	1	NA	NA		4.38	1	NA	NA		62.7	1	NA	NA	

[a] degrees of freedom

[b] sum of squares

[c] p-value

[d] "/" = the nesting term; Plate = the 96-well plate on which samples were homogenized from which they were dilution plated

[e] "*" = the interaction term

Table S9 PERMANOVA results for CFU data corresponding to Figure S9

	Unrarefied Bray Curtis (Absolute abundance)						Rarefied Bray Curtis (Relative abundance)				
	Df[a]	SS[b]	R^2	F	p[c]		SS	R^2	F	p	
Source (apples or compost)	1	0.31	0.01	1.54	0.16		0.00	0.00	0.08	0.78	
Fly sex	1	1.20	0.05	5.98	0.001	***	0.19	0.03	3.72	0.06	.
Experimental replicate	1	3.71	0.17	18.44	0.001	***	2.37	0.38	47.52	0.001	***
*So * Se [d]*	1	0.15	0.01	0.77	0.57		0.10	0.02	2.04	0.16	
Residual	82	16.50	0.75				3.59	0.57			
Total	86	21.87	1.00				6.26	1.00			

[a] degrees of freedom

[b] sum of squares

[c] p-value

[d] "*" = the interaction term

Table S10. Sampling site details

CITY	ORCHARD NAME	LATITUDE	LONGITUDE	SEPARATE COMPOST COLLECTION	# LINES	DATE COLLECTED	FRUIT TYPE
CHARLOTTESVILLE, VA	Carter Mountain Orchard	38.07474	-78.38155		17	10/20/21	Apple
CHURCHVILLE, MD	Lohr's Orchard	39.539	-76.2399		12	10/21/21	Apple
MEDIA, PA	Indian Orchards, Linvilla Orchards	39.8845	-75.4125		28	10/28/21	Apple
MIDDLEFIELD, CT	Lyman Orchards	41.49383	-72.71145	Y	13	10/1/21	Apple; separately, peaches and pears
HARVARD, MA	Westward Orchards	42.50867	-71.57071	Y	10	9/30/21	Apple
DURHAM, NH	Demeritt Hill Farm	43.08745	-71.04666		24	9/30/21	Apple
BOWDOIN, ME	Rocky Ridge Orchard	44.0253	-69.94379		19	9/29/21	Apple
ETNA, ME	Conant Orchards	44.82082	-69.11144	Y	~20	9/29/21	Apple
SANTAQUIN, UT	Rowley's Red Barn	39.966865	-111.791562		na	10/10/20	Peach
LINDON, UT	Martel Famrs	40.3327707	-111.7219739		na	9/29/20, 10/6/20, 10/10/20	Peach
ALPINE, UT	Burgess Orchards	40.4457589	-111.7818936		na	10/9/20	Peach
OGDEN, UT	Stake Farm North Ogden Peach Orchard	41.326252	-112.011375		na	10/9/20	Peach
LOGAN, UT	Zollinger Fruit and Tree Farm	41.72573	--111.809883		na	10/8/20	Peach

Table S11 Sampling site details for individual isofemale lines

LINE NAME	ORCHARD	LATITUDE	LONGITUDE	DATE COLLECTED	FRUIT TYPE
**TW**	Conant Orchards	44.82082	-69.11144	10-26-2023	Apple Δ[a]
**TI**	Conant Orchards	44.82082	-69.11144	10-26-2023	Apple Δ
**ER**	Conant Orchards	44.82082	-69.11144	10-26-2023	Apple ❍ [a]
**P19**	Conant Orchards	44.82082	-69.11144	9-30-2023	Compost ☐ [a]
**P20**	Conant Orchards	44.82082	-69.11144	9-30-2023	Compost ☐
**P21**	Conant Orchards	44.82082	-69.11144	10-26-2023	Compost ☐

[a] symbol position shown in Figure 5A

Figure S1. The relationship between microbiota composition and latitude in flies sampled from Utah in Fall 2020. A) Sampling map. B) Taxon plot. Plots showing the correlation between Bray Curtis distance (a measure of beta-diversity in microbiota composition) and environmental distance defined by C) latitude, and D) temperature. Mantel test results rho (ρ) and p-value (p) are also shown. Read counts were rarefied to 500 reads prior to analysis.

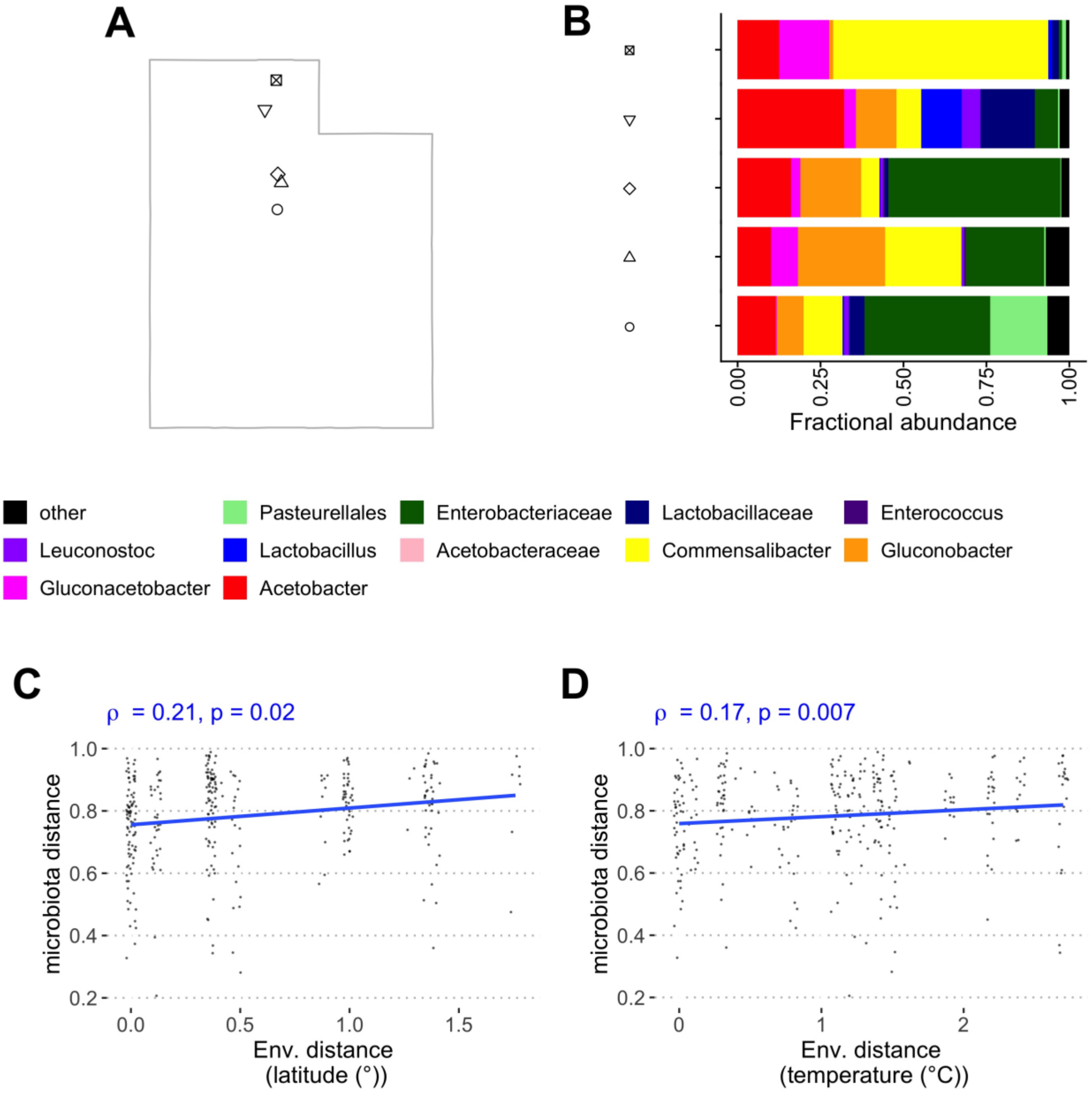

Figure S2. Principal coordinate ordination of Bray-Curtis distances for microbiota from different samplings in the eastern USA. *Data correspond to Figure 1A-E*. A) A map of sampling locations and symbols. Samples were collected from the eastern USA in B) 2009 (from apples and peaches), C) 2018, D) 2018 (from apples), E) 2021 (from apples).

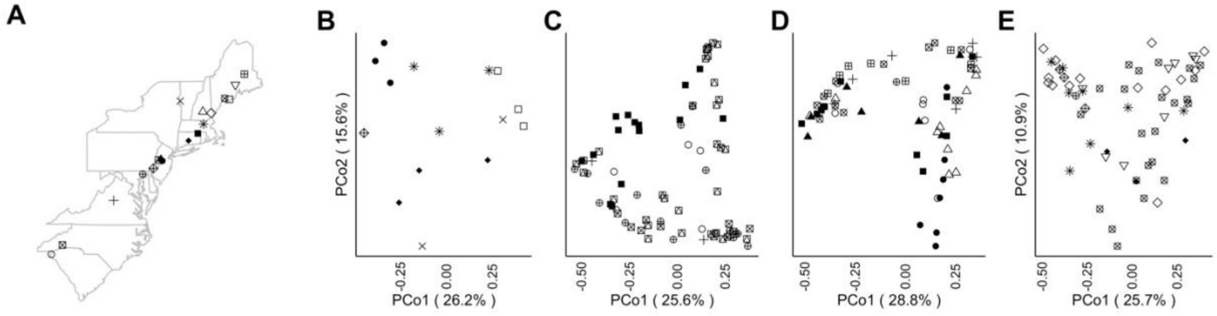

Figure S3. The correlation between microbiota composition and environmental distance. *Data correspond to Figure 1B-M*. Samples were collected from the eastern USA in A,E,I,M) 2009 (from apples and peaches), B,F,J,N) 2018 (from grapes), C,G,K,O) 2018 (from apples), D, H, L, P) 2021 (from apples). Environmental distance was defined as shown on each x-axis. Microbiota distance was defined using the A-H) weighted or I-P) unweighted Unifrac distance metric.

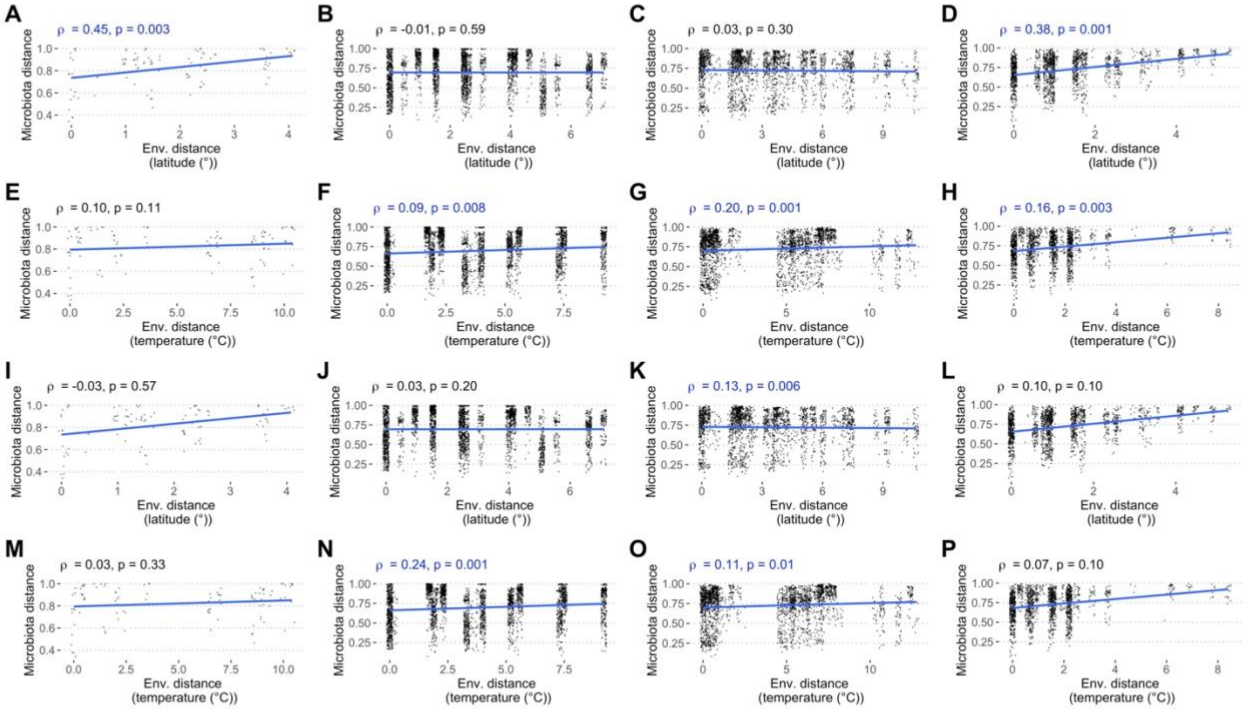

Figure S4. The relationship between A-E) daily maximum temperature and latitude or F-J) daily maximum UV irradiance (UVI) and relative humidity (RH) during various sampling periods. Fly sampling sites were from A,F) 2009, B,G) 2018 in vineyards, C,H) 2018 in orchards, D,I) 2021 from apples in the eastern USA, and E,J) 2020 from peaches in Utah. Results are calculated from metadata used to calculate Table S1. Significant correlations were tested using a Pearson's correlation test, which was less statistically conservative than a Spearman's rank correlation test. Regardless of the test that was used, the only sites with significant correlations were orchards sampled in 2018.

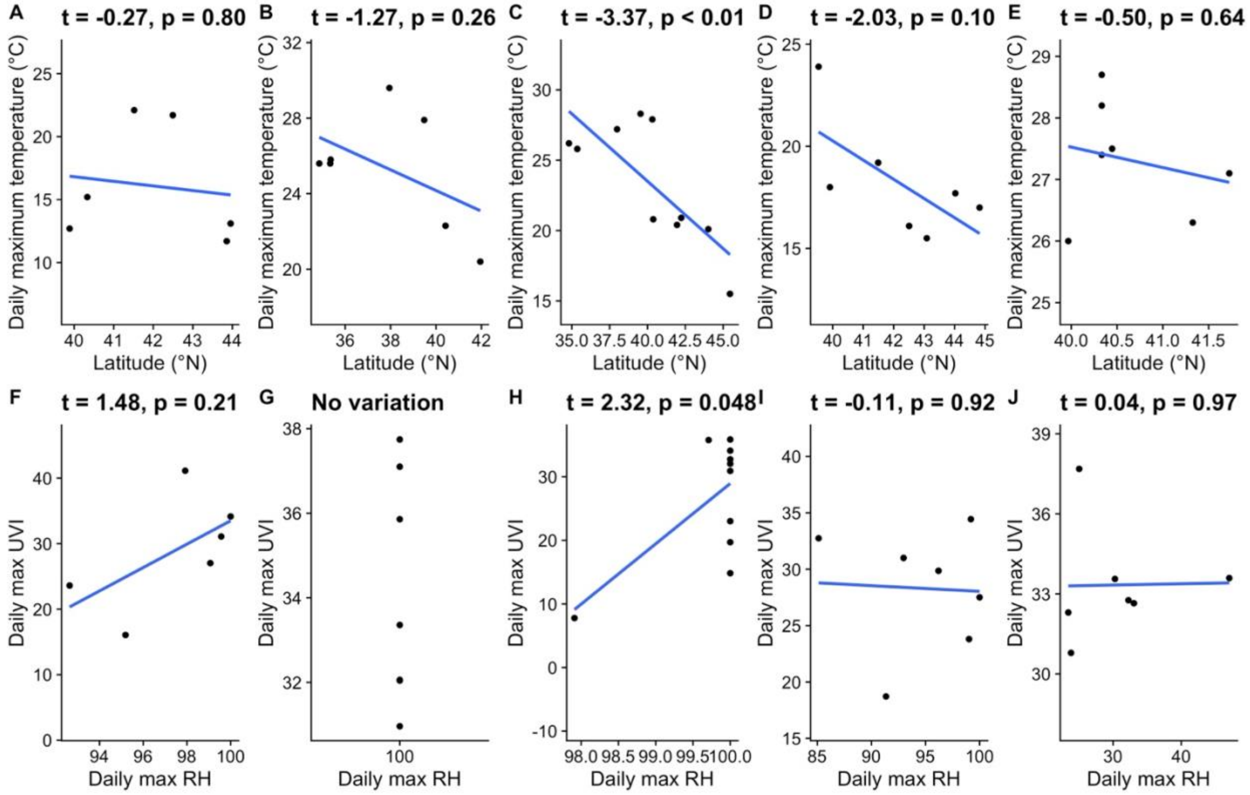

Figure S5. Temperature-dependent variation in the microbiota composition of common garden fly populations, collected at the locations shown on the y-axis (see TABLE S10 for code explanation) and reared in the laboratory under 6-species gnotobiotic conditions. CFU counts of AAB (red) and LAB (blue) recovered from homogenized pools of 2 flies each are shown. Data for 4-6 day old A,C,E,G,I,K) male and B,D,F,H,J,L) female flies moved from 25°C to A,B,G,H) 15°C, C,D,I,J) 25°C, or E,F,K,L) 32°C for 3 days immediately prior to homogenization are shown. Relative abundances are shown as the mean of AAB counts divided by the mean of LAB counts, with the fraction of LAB shown as a white point and the overlayed violin plot showing the distribution of fractional LAB abundance. Significant differences in relative abundances of LAB were determined by PERMANOVA (TABLE S2). Absolute CFU abundances are shown as the mean and standard error of the mean of all replicates. Significant differences in AAB and LAB abundance were determined by a Kruskal-Wallis test with a post-hoc Dunn test, and different letters over (AAB) or under (LAB) the bars report significant differences in their abundance

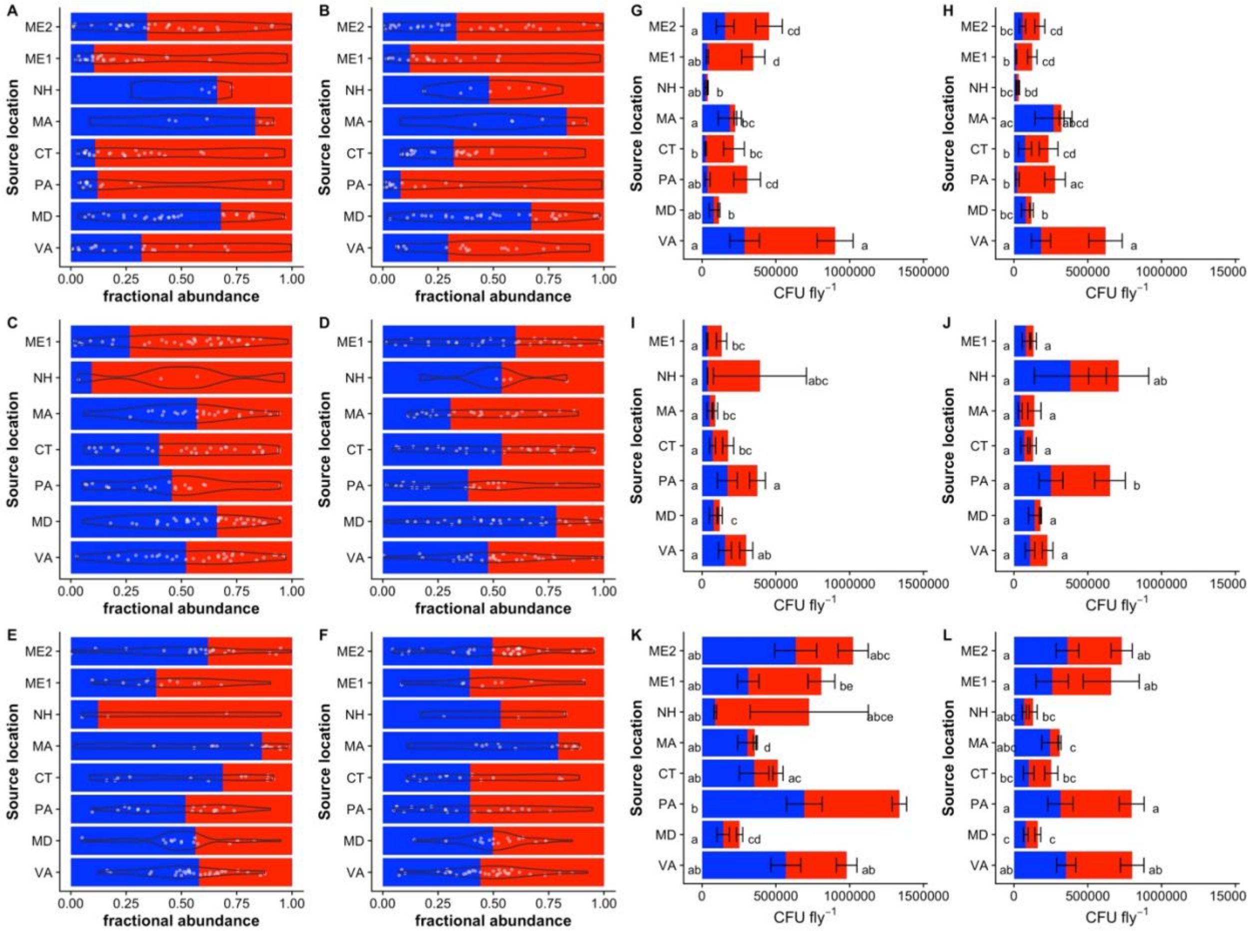

Figure S6. Photoperiod-dependent variation in the microbiota composition of common garden fly populations, collected at the locations shown on the y-axis (see TABLE S10 for code explanation) and reared in the laboratory under 6-species gnotobiotic conditions.

The relative A) and absolute B) abundances of AAB (red) and LAB (blue) colony forming units (CFUs) in flies reared at varying photoperiods are shown. Relative abundances are shown as the mean of AAB counts divided by the mean of LAB counts, with the fraction of LAB shown as a white point and the overlayed violin plot showing the distribution of fractional LAB abundance. Significant differences in relative abundances of LAB were determined by PERMANOVA (TABLE S2). Absolute CFU abundances are shown as the mean and standard error of the mean of all replicates. Significant differences in AAB and LAB abundance were determined by a Kruskal-Wallis test with a post-hoc Dunn test, and different letters over (AAB) or under (LAB) the bars report significant differences in their abundance.

The same data are shown, divided by sex and photoperiod condition, as CFU counts of AAB (red) and LAB (blue) recovered from homogenized pools of 2 flies each are shown. Data for 4-6 day old C,E,G,I,K,M) male and D,F,H,J,L,N) female flies moved from a 12h light:dark cycle to C,D,I,J) 1h light:23 h dark, E,F,K,L) 12h light:dark, or G,H,M,N) 23 h light: 1 hr dark cycle for 3 days immediately prior to homogenization are shown. Relative abundances are shown as the mean of AAB counts divided by the mean of LAB counts, with the fraction of LAB shown as a white point and the overlayed violin plot showing the distribution of fractional LAB abundance. Significant differences in relative abundances of LAB were determined by PERMANOVA (TABLE S2). Absolute CFU abundances are shown as the mean and standard error of the mean of all replicates. Significant differences in AAB and LAB abundance were determined by a Kruskal-Wallis test with a post-hoc Dunn test, and different letters over (AAB) or under (LAB) the bars report significant differences in their abundance

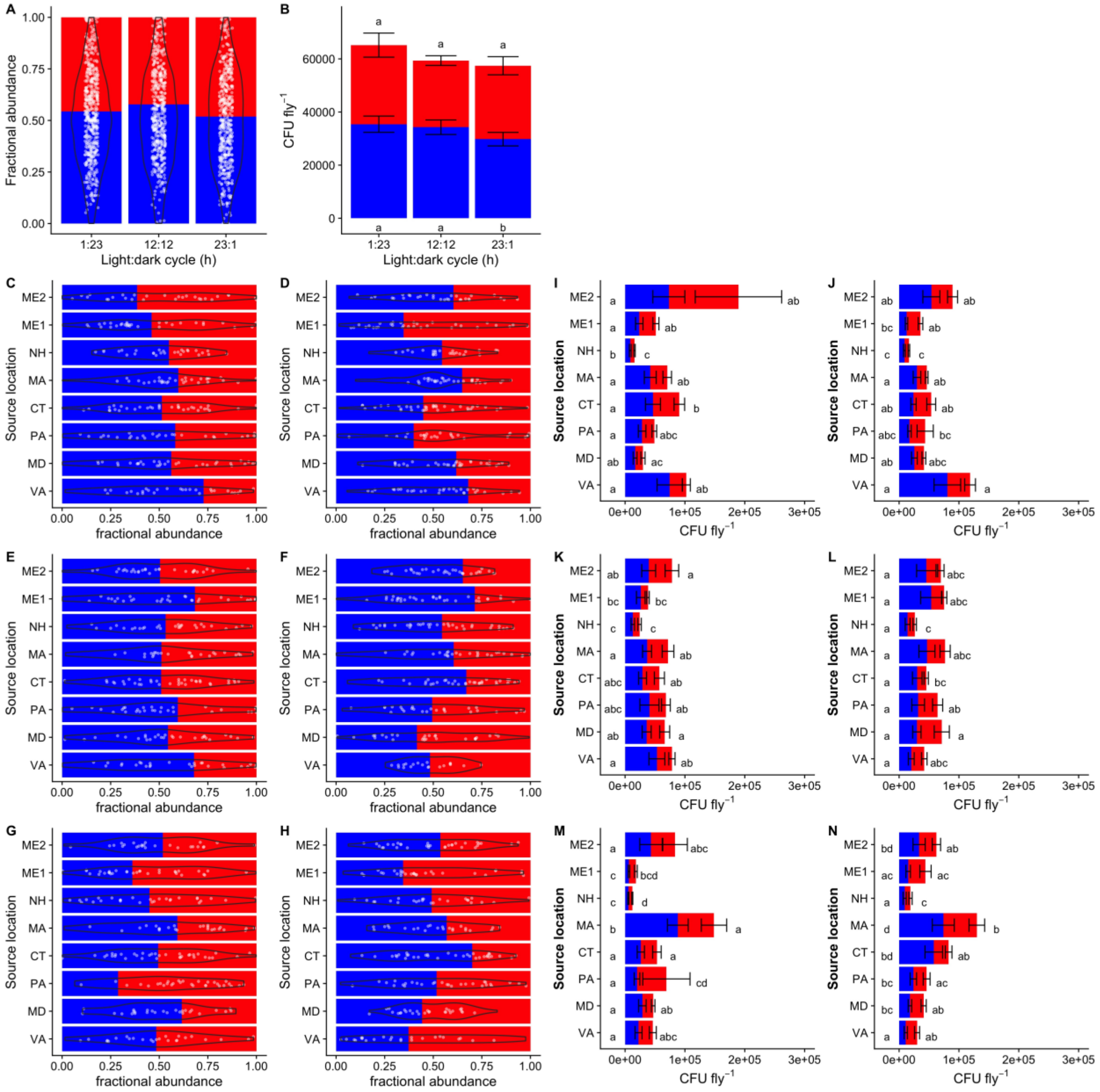

A
B
C
D
E
F
G
H
I
J
K
L
M
N
Fractional abundance
1.00
0.75
0.50
0.25
0.00
1:23
12:12
23:1
Light:dark cycle (h)
CFU fly^{-1}
60000
40000
20000
0
a
a
a
a
a
b
Source location
ME2
ME1
NH
MA
CT
PA
MD
VA
fractional abundance
0.00
0.25
0.50
0.75
1.00
0e+00
1e+05
2e+05
3e+05

Figure S7. The total and resident *D. melanogaster* microbiota composition is distinct from the diets of wild flies. *Data corresponds to Figure 2A,D*. The 16S rRNA V4 region was sequenced in samples of individual wild *Drosophila melanogaster*, their diets, and nearby soil locations from A) multiple locations in the eastern USA. B) Flies were immediately frozen after collection ('Flies (total)') or starved in empty vials for > 2 h ('Flies (resident)') after transient microorganisms had passed through the fly gut with the bulk flow of diet. Fruit and soil samples were also taken, and each row indicates a single quartet of the total microbiota of a fly, resident microbiota of a fly, the fruit it was collected from, or an immediately adjacent soil sample. White spaces either separate sampling sites, or are 'blanks' where a sample was either not obtained or insufficient reads were recovered upon sequencing. C) Bars are the averages of multiple samples (N = mean 6.5 ± sem 1.2, min = 3, max = 10 samples per bar condition) rarefied to 845 reads each. D) Bray Curtis distances between samples assigned to the groups in the Reference and Comparison rows, including the mean distance between samples (blue bar). Different letters over the clusters of points represent statistically significant differences in distance between the comparison groups as determined by a Kruskal-Wallis test with a post-hoc Dunn test.

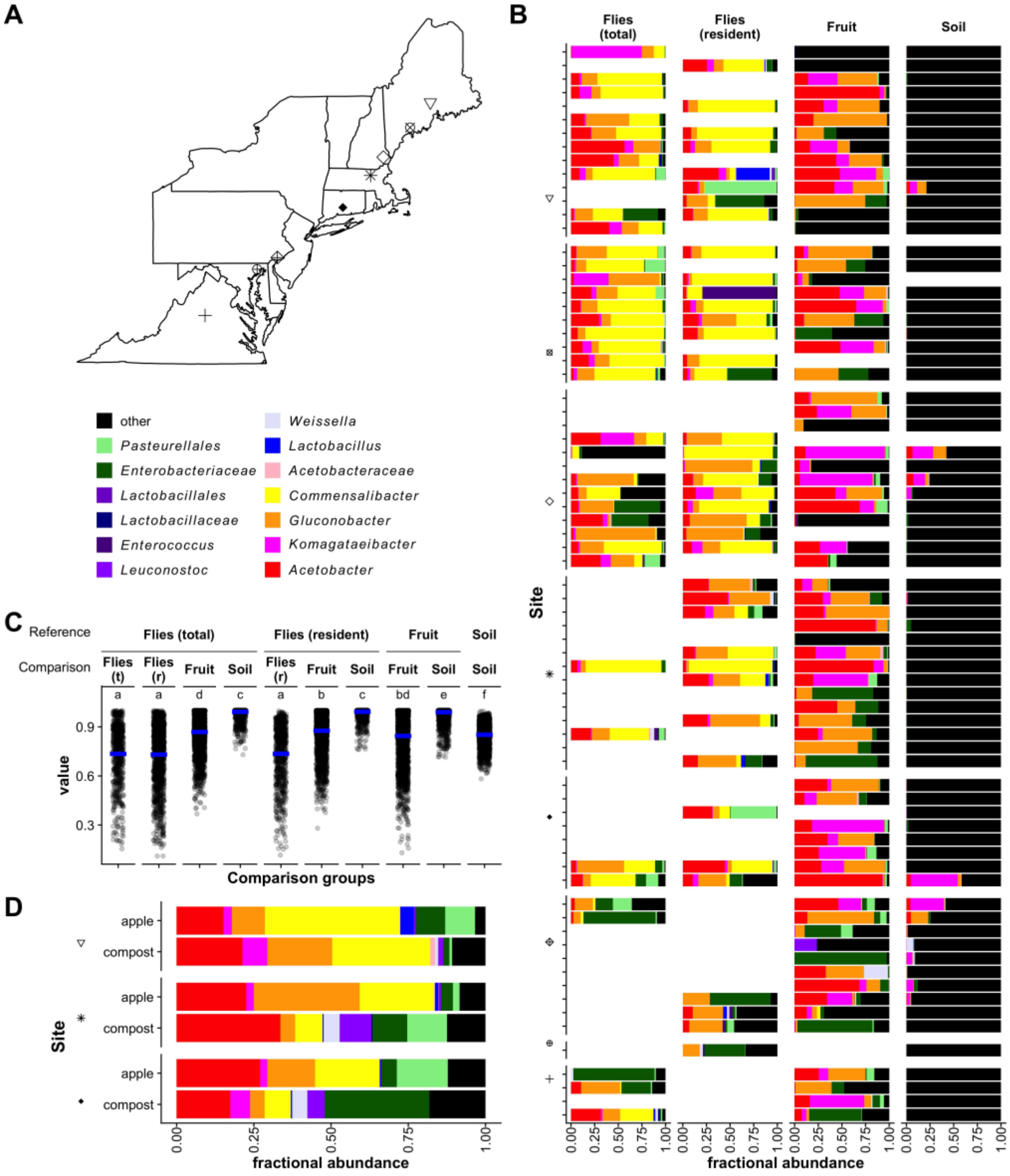

Figure S8. Correlation between microbiota composition and time. *Data correspond to Fig. 4B,I.* Correlation plots showing the data underlying Mantel tests that comparing the difference in time between samplings and the microbiota distance of the samplings from individual piles of peaches and apples established at different times over a fall season. Microbiota distance was defined using A-B) unweighted Unifrac and C-D) Bray Curtis distance metrics. The difference in time was calculated from A,C) calendar date (as in Fig. 4B) or B,D) the time each pile was established (as in Fig. 4H). Black points show the distances between two samplings, and a trendline is shown in blue.

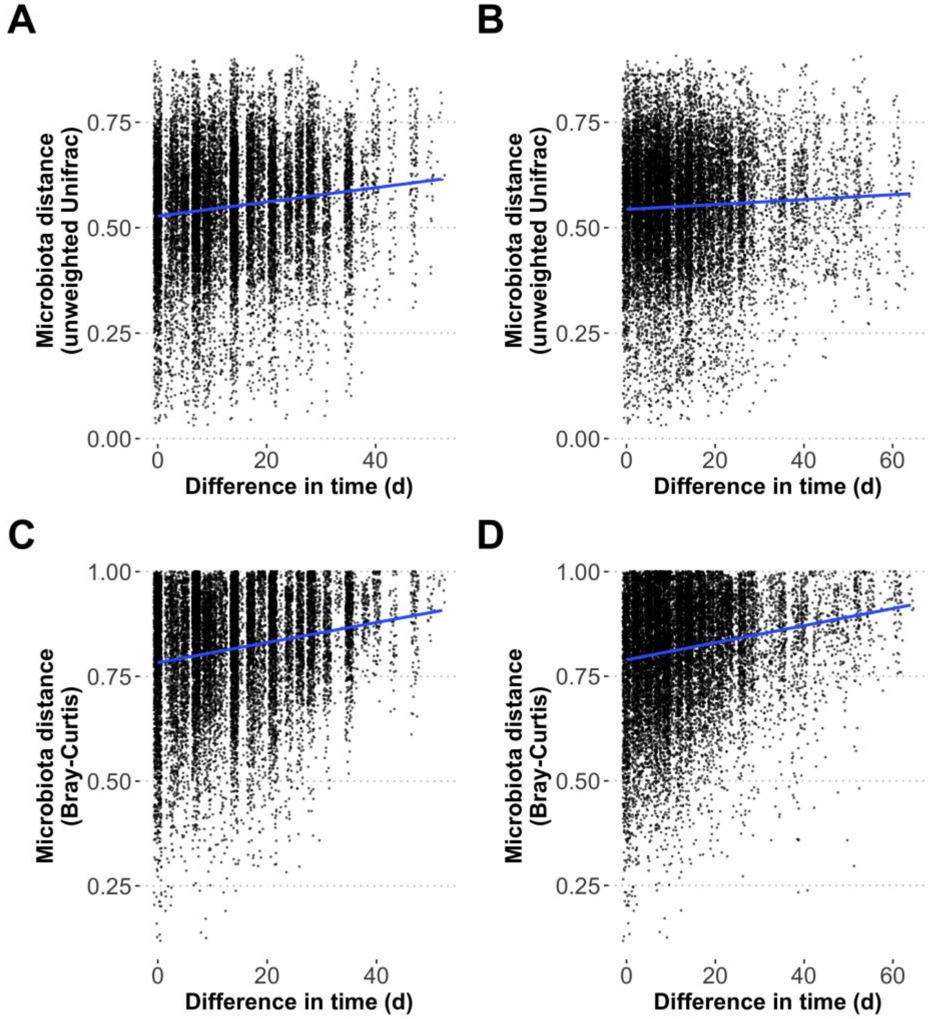

Figure S9. Microbiota composition in gnotobiotic 6-species isofemale lines. A-B) Male flies, C-D) female flies, A,C) absolute CFU counts, B,D) relative abundance. AAB CFUs (red) and LAB CFUs (blue) are shown. Different letters over or under the bars show significant differences by a Wilcoxon test (there are no differences). See Table S9 for PERMANOVA results that correspond to the data.

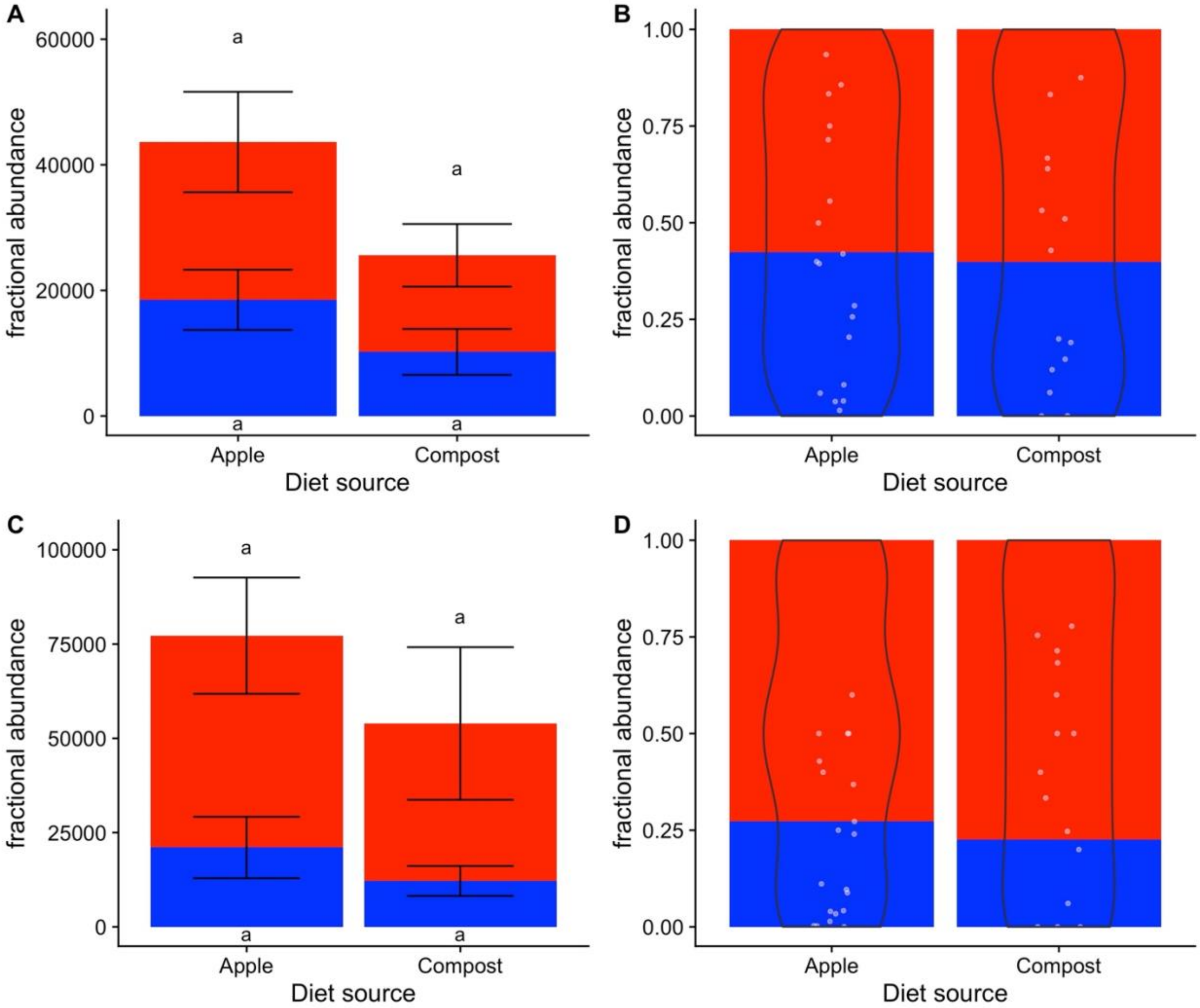

Figure S10. Variation in microbiota composition with *Wolbachia* positivity in flies sampled from different fruits at an orchard in Middlefield, CT. A) Taxon plot. ANCOM revealed significant differences in the abundance of B) *Acetobacteraceae* reads when clustered at the family level, or C) a specific *Commensalibacter* ASV when considered at the ASV level, between *Wolbachia*-negative and *Wolbachia*-positive flies

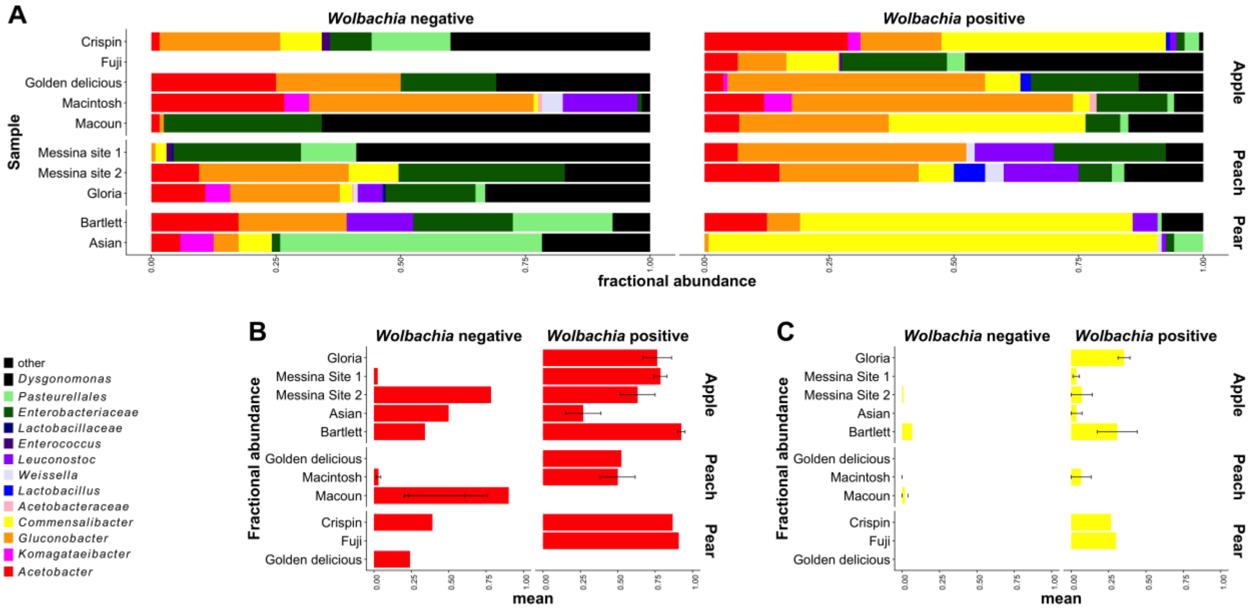

Supplement: Supplemental material — Tables S1 to S11; Fig. S1 to S10. [file aem.00883-25-s0002.pdf]
